# Products, Performance, and Technological Development of Ambulatory Oxygen Therapy Devices: Scoping Review

**DOI:** 10.2196/81077

**Published:** 2026-01-27

**Authors:** Shohei Kawachi, Mariana Hoffman, Lorena Romero, Magnus Ekström, Jerry A Krishnan, Anne E Holland

**Affiliations:** 1 Respiratory Research@Alfred School of Translational Medicine Monash University Melbourne, Victoria Australia; 2 Department of Rehabilitation Shinshu University Hospital Matsumoto, Nagano Japan; 3 Institute for Breathing and Sleep Melbourne, Victoria Australia; 4 Ian Potter Library The Alfred Hospital Melbourne, Victoria Australia; 5 Respiratory Medicine, Allergology and Palliative Medicine Department of Clinical Sciences Lund Lund University Lund Sweden; 6 Breathe Chicago Center University of Illinois Chicago Chicago, IL United States; 7 Departments of Physiotherapy and Respiratory Medicine Alfred Health Melbourne, Victoria Australia

**Keywords:** ambulatory oxygen therapy, automatic titration of oxygen, home oxygen therapy, liquid oxygen, medical device innovation, oxygen cylinder, portable oxygen concentrator, pressure swing adsorption, zeolite

## Abstract

**Background:**

Ambulatory oxygen therapy is prescribed for patients with chronic lung diseases who experience exertional hypoxemia. However, available devices may not adequately meet user requirements, and their performance characteristics are heterogeneous.

**Objective:**

This study aims to identify devices available for delivery of ambulatory oxygen therapy, the technologies that they use to generate oxygen, the performance characteristics of each device, and the development status.

**Methods:**

We used medical and engineering databases to identify peer-reviewed papers (eg, MEDLINE, IEEE). Gray literature was used to identify additional descriptions of ambulatory oxygen devices in military medicine, space exploration, or patents. The last search was conducted in September 2025. Documents that described a device that can deliver oxygen in an ambulatory context (defined as weighing less than 10 kg) and were written in English were included. Search results were screened for inclusion by 2 independent reviewers. Data were synthesized by descriptively mapping the performance of each product, the technology used, and the development status of emerging technologies.

**Results:**

From 9702 records identified, a total of 166 met eligibility criteria (106 scientific publications and 60 gray literature). We identified 33 portable oxygen concentrators (POCs; 29 commercially available), 10 oxygen cylinders, and 6 portable liquid oxygen (LOX) devices. The POC products showed a trade-off between portability and oxygen delivery capacity (maximum flow rate ranging from 2.0 to 6.0 L/min; device weight ranging from 1.0 to 9.1 kg). Pressure swing adsorption with zeolite was the most common oxygen generation technology in POCs on the market. The mean maximum continuous operating time of POCs was 3.8 hours. Two prototype POCs (maximum flow rate of 4-6 L/min and device weight of 8-9 kg) were developed for space exploration using modified adsorbents. LOX devices were the lightest and had the longest continuous operating time. Innovations in delivery included the downsizing of a POC by using nanozeolite as an adsorbent and pulse oximeter oxygen saturation (SpO_2_)–targeted automatic titration of oxygen delivery based on the user’s SpO_2_.

**Conclusions:**

This scoping review is the first study to integrate medical, engineering, and gray literature on ambulatory oxygen devices and their development. Although prior literature has narratively explained the products and technologies, no previous research has systematically investigated them. This review showed that POCs available to consumers may not meet the needs of patients in terms of flow rate, portability, and operating time. LOX devices offered superior performance but are limited by high costs. Limitations of this review include the difficulty of comparing product performance across oxygen delivery settings and that the records were largely obtained from English-language sources. Innovation in ambulatory oxygen technology has been limited over the past decade, highlighting urgent need for research and development of new lightweight devices with higher oxygen delivery.

**Trial Registration:**

OSF Registries 10.17605/OSF.IO/QS7FX; https://osf.io/qs7fx

## Introduction

### Background

Supplemental oxygen may be prescribed to correct severe hypoxemia at rest, with exertion, and/or during sleep. Ambulatory oxygen therapy is defined as the use of supplemental oxygen during exertion [1]. The American Thoracic Society guideline conditionally recommends prescribing ambulatory oxygen therapy for patients who have severe exertional room air hypoxemia [1], based on acute improvements in exercise capacity and modest evidence of improvements in health-related quality of life [2,3]. Portable oxygen concentrators (POCs), oxygen cylinders, and liquid oxygen (LOX) can be used to deliver ambulatory oxygen therapy [1]. Oxygen cylinders for ambulatory use can deliver continuous oxygen flow up to 6 L per minute but run out quickly, especially when used at high flow rates, and need to be refilled. POCs supply concentrated oxygen by removing nitrogen from the air as long as they have a power source; however, they may provide oxygen only intermittently (“pulse flow” triggered by inspiration) and may have limited battery life. In continuous-flow settings, oxygen is delivered throughout both inhalation and exhalation, resulting in substantial oxygen waste during exhalation. Pulse-flow settings were developed to minimize this waste by supplying oxygen only during inspiration, thereby conserving both oxygen and battery power [4]. However, if the pulse is not synchronized well with inspiration, a portion of the oxygen bolus may be exhaled before reaching the alveoli [5]. LOX supplies oxygen by gradually evaporating LOX at cryogenic temperatures and can deliver higher flow rates for longer periods. However, LOX devices are costly, and availability may be limited [6].

In previous studies, users of portable oxygen devices reported a lack of physically manageable portable systems, a lack of devices capable of delivering higher oxygen-flow rates (>3 L/min), and an inability to leave their homes for more than 2-4 hours due to lack of reliable and enduring ambulatory oxygen supply [7-9]. In addition, users may face large out-of-pocket expenses for the ongoing costs of oxygen equipment [7]. Given these limitations, people using ambulatory oxygen reported that it was important to have a variety of device options that allowed a choice based on their individual needs [9]. However, there is little information on the types of devices available, the performance of each device, and the costs of ambulatory oxygen therapy devices [7]. It is possible that technologies to deliver ambulatory oxygen therapy are available in other fields (eg, military medicine) that are not yet available as commercial ambulatory devices.

A scoping review was deemed most appropriate to investigate the current status of portable oxygen devices, as the concepts of interest (technology, performance characteristics, and cost) extend beyond the medical field and may be published outside traditional peer-reviewed medical literature (eg, patent documents, technical reports) [10]. This scoping review aimed to identify the range of available portable oxygen devices, the technologies that are used to generate oxygen by the devices, the performance of each device, and the costs associated with its use. In addition, given the limitations of current portable oxygen devices, it is clinically important to clarify the status of technological innovation. Some patients require high oxygen flow rates that exceed the capacity of the currently available portable oxygen devices [11].

Furthermore, it is recognized that the weight of portable oxygen systems may limit their usability in clinical practice [12]. This may reduce adherence and contribute to inconsistent evidence for efficacy in exertional hypoxemia [11,13]. Therefore, we also aimed to identify innovations in the design of ambulatory oxygen therapy devices, such as improvements in weight or flow rates, and pulse oximeter oxygen saturation (SpO_2_)–targeted automatic titration of oxygen delivery, which may not be available commercially [14].

### Objectives

This scoping review aimed to identify the range of available portable oxygen devices, the technologies that are used to generate oxygen by the devices, the performance of each device, and the costs associated with its use. We also aimed to identify innovations in the design of ambulatory oxygen therapy devices, such as improvements in weight or flow rates and SpO_2_-targeted automatic titration of oxygen delivery, which may not be available commercially.

## Methods

### Overview

This scoping review was conducted according to the PRISMA-ScR (Preferred Reporting Items for Systematic Reviews and Meta-Analyses extension for Scoping Reviews) guideline (Multimedia Appendices 1-3; [15]), and the Joanna Briggs Institute (JBI) Manual for Evidence Synthesis [16]. The protocol was registered prospectively with the Open Science Framework on May 29, 2024.

### Eligibility Criteria

We included English language documents that described a device that can deliver oxygen in an ambulatory context, including scientific papers (including review articles) and gray literature (conference proceedings, patent documents, company websites, technical reports, and government documents). We excluded documents that only described stationary equipment, high-flow nasal oxygen therapy, hyperbaric oxygen therapy, positive pressure ventilation systems, heliotherapy, short-burst oxygen therapy, and wall-based high-flow nasal oxygen therapy, as well as those limited to over-the-counter medical devices.

### Search Strategy

An initial search was performed on May 29, 2024, in MEDLINE (Ovid) and IEEE Xplore. After the initial search, the text words in the titles and abstracts of the retrieved articles and the index terms used to describe the articles were analyzed to refine the search terms. For the second search using updated terms, we searched scientific papers in 5 databases (MEDLINE [Ovid], Embase [Ovid], SCOPUS, CENTRAL [Wiley], and IEEE Xplore) using the search strings created with index terms (MeSH [Medical Subject Headings] terms or IEEE terms). Search strings are shown in Table S1 in Multimedia Appendix 4.

For searching gray literature, the following three strategies were used based on the guideline for gray literature and a previous study [17,18]: (1) targeted website browsing and searching, (2) gray literature database search, and (3) search engine searching. Specifically, technical reports, white papers including military medicine, and other gray literature on portable oxygen devices (manuals, company websites on product performance, etc) were searched using the appropriate websites respectively (National Technical Reports Library, World Health Organization, Defense Technical Information Center, International Health technology Assessment Database, and Google Advanced). As complex search strings were not allowed in some gray literature databases, we adjusted the search strings to match the database functionality. Gray literature search strings are also shown in Table S1 in Multimedia Appendix 4. Google Advanced searches were run on corporate, government, and military domains (.com, .gov, and .mil) to review the top 100 results from each. The latest patents of inventions and developments for the period January 1, 2022, to June 25, 2024, were searched in the World Intellectual Property Organization (WIPO) database using adjusted search strings. The search was repeated on September 29, 2025, for CENTRAL, MEDLINE (Ovid), and Embase (Ovid), on September 25, 2025, for all other databases including Scopus, IEEE Xplore, and gray literature sources, and on September 29, 2025, for WIPO.

A third search consisted of hand searching reference lists of all selected articles and review papers to identify any additional articles not found by the first two search methods. Additionally, Google Advanced searches were performed to obtain missing product performance information. The search period in each database was 2004 to the present to capture devices that were currently or recently available. We reported database searches and literature selection according to PRISMA-S (Preferred Reporting Items for Systematic Reviews and Meta-Analyses Search extension) [19].

### Selection of Studies

Scientific papers were imported from the databases into the Covidence platform [20], and gray literature search results were imported into a spreadsheet. In the Covidence platform, duplicate records were automatically removed. Two independent reviewers (SK and MH) screened titles and abstracts of scientific papers for eligibility. Studies that met the inclusion criteria, or for which eligibility was unclear, underwent full-text review. Gray literature records were also screened for eligibility by 2 independent reviewers (SK and MH). Disagreements in study selection were resolved by consensus or by consulting a third reviewer (AEH). The reasons for exclusion at the full-text stage are reported.

### Outcomes of Interest

Outcome selection was guided by patient priorities for oxygen devices, identified in previous studies and are shown in Table 1 [7-9].

**Table 1:** Outcomes of interest in the scoping review of ambulatory oxygen therapy devices extracted from scientific and gray literature published between 2004 and 2025.

Category	Outcome
Portable oxygen devices and their performance characteristics	Type of device (POCs^a^, portable oxygen cylinders, portable LOX^b^) Product name Manufacturer Release date Size of device Weight of device (and battery weight where relevant) Pulse or continuous flow Method of transporting the device Range of flow rates in the pulse flow setting Range of flow rates in continuous flow setting Pulse-dose bolus volume in maximum pulse flow setting (as much as possible at a respiratory rate of 20 breaths per minute) Maximum continuous operating time (in the case of POC, battery duration, and in the case of oxygen cylinders or LOX, the duration until the oxygen stored in the tank runs out) Concentration of supplied oxygen^c^ Operating noisec Battery recharge time Trigger sensitivity Technology used to deliver oxygen (eg, nature of the sorbent in POC) Product availability to consumers FDA^d^ and EMA^e^ approved or equivalent FAA^f^ approved for air travel or equivalent Frequency of product failures Remote control function for changing flow rate
Cost of portable oxygen devices	Description of publicly available supplier costs for each device (eg, purchase and rental costs of equipment, electricity costs for its use, etc)^g^

^a^POC: portable oxygen concentrator.

^b^LOX: liquid oxygen.

^c^Concentration of supplied oxygen and operating noise were included as they are important for the International Organization for Standardization (ISO) requirements to be approved as a medical device through the United States Food and Drug Administration and European Medicines Agency [21].

^d^FDA: Food and Drug Administration.

^e^EMA: European Medicines Agency.

^f^FAA: Federal Aviation Administration.

^g^The cost information, only officially disclosed by the company of each product, was extracted.

### Data Charting Process

Data from the included sources of evidence were charted using a custom-designed form (SK and MH). Two review authors (SK and MH) independently charted the data from the eligible studies. Disagreements regarding the data charting between authors were resolved by discussion. If consensus could not be reached, a third author (AEH) reviewed the study and arbitrated.

The data chart included: types of publication (scientific paper, patent documents, technical report, etc), types of content (products, performances, costs, etc), author, year of publication, country where the information originated, information related to the products and performances outlined, and information related to the costs.

### Risk of Bias (Quality Assessment)

Scoping reviews are conducted to provide an overview of the existing evidence regardless of methodological quality or risk of bias [22]. Therefore, the included sources of evidence are typically not critically appraised for scoping reviews. As such, we did not undertake a quality assessment of the included sources of evidence.

### Data Synthesis

To synthesize data on products and their performance, we prioritized information published by the product company (including user manual), scientific papers, white papers, technical reports, and websites, in that order, if there was different information on the same item regarding one product. If multiple products were identified as having the same product name but differing only in numbers, the newest product was checked at the official website, and only the newest one was extracted (eg, Eclipse 1/2/3/4/5 Caire, Ball Ground, United States). As stationary oxygen concentrators are generally considered to weigh 10 kg or more, and POCs range from 1-9 kg [4], we defined ambulatory oxygen devices in this study as those weighing less than 10 kg and excluded heavier devices. Performance characteristics, the technology used, and the current status of development were reported descriptively. Costs for ambulatory oxygen therapy were mapped descriptively by country and type of device (portable oxygen cylinders, POCs, and portable LOX). Descriptive statistics were used to describe all data. Categorical data were presented as frequency and percentages. In addition to descriptive synthesis, we developed a gap map to describe areas in need of future development. The gap map addressed the degree to which key patient needs for ambulatory oxygen devices (oxygen flow rate, device weight, operating time, and auto-titration) are met by characteristics of current products or those in development. Patients’ needs extracted from previous studies were used as the framework [7-9].

## Results

### Principal Findings

**Figure 1:**
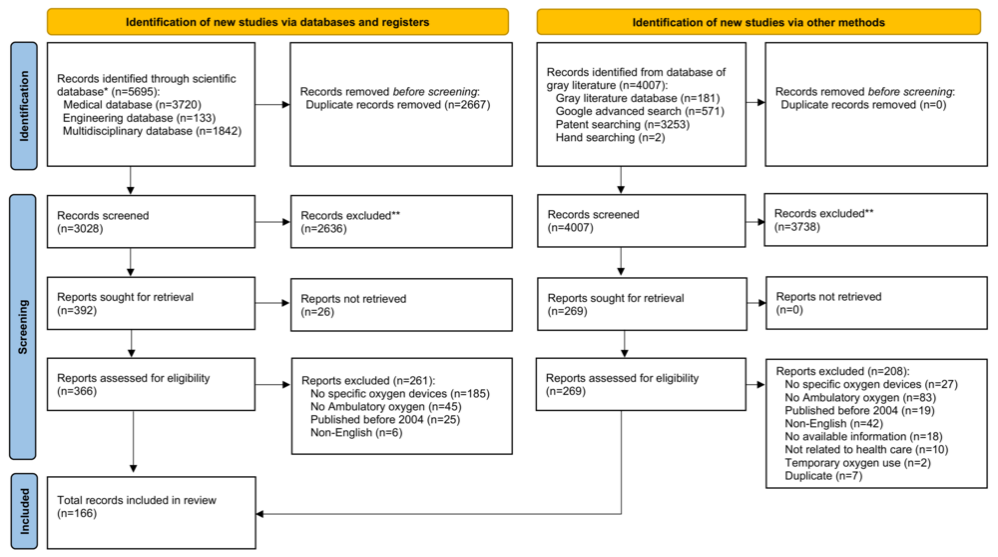
PRISMA (Preferred Reporting Items for Systematic Reviews and Meta-Analysis) flow diagram showing the identification, screening, and inclusion of studies in this scoping review of ambulatory oxygen therapy devices. The search included scientific and gray literature from 2004 to 2025 across medical, engineering, military, and space exploration fields.

A total of 9702 records were identified from scientific databases (medical database: n=3720; engineering database: n=133; multidisciplinary database: n=1842), and 4007 records from gray literature. After removing duplicates, a total of 3028 records from scientific databases and 4007 records from gray literature sources were screened (Figure 1). Of these, a total of 166 records related to ambulatory oxygen therapy devices were finally included in this review (n=106 from scientific databases; n=60 from gray literature sources). Among the 166 included records, a total of 81 (48.8%) originated from North America, 37 (22.3%) from Europe, 31 (18.7%) from Asia, 11 (6.6%) from Oceania, 2 (1.2%) from Africa, and 2 (1.2%) from South America (Table S2 in Multimedia Appendix 4). The scientific literature included 85 original research articles, 20 review articles (of which 1 was a systematic review and 19 were narrative reviews), and 1 clinical guideline. The gray literature comprised 7 technical reports, 3 white papers, 3 clinical trial registrations, 14 government-related websites (eg, military, energy, and space exploration), 4 company websites, 1 charitable organization website, and 28 patents (Tables 2 and 3). Although we identified 3 chemical oxygen generators in the scientific literature, they were excluded because chemical oxygen generators exhaust in 30 minutes or less and their output cannot be adjusted, making these devices unsuitable for delivery of ambulatory oxygen in clinical care [23].

**Table 2:** Summary of literature on ambulatory oxygen therapy devices (n=166). Types of sources identified in this scoping review include scientific articles, technical reports, patents, government and military documents, and company websites published between 2004 and 2025.

Type of literature				Records, n (%)
**Scientific literature (n=106)**				
	Original paper			85 (51)
	**Review paper**			20 (12)
		Systematic	1 (1)	
		Narrative	19 (11)	
	Guideline			1 (1)
**Gray literature (n=60)**				
	Technical report			7 (4)
	White paper			3 (2)
	Clinical trial registry			3 (2)
	**Government website**			14 (9)
		Military	8 (5)	
		Energy	5 (3)	
		Space exploration	1 (1)	
	Company website			4 (2)
	Charitable organization			1 (1)
	Patent			28 (17)

**Table 3:** Summary of content from eligible literature (n=166) included in this scoping review of ambulatory oxygen therapy devices. Information sources span medicine, engineering, energy, military, and space exploration fields and describe portable oxygen concentrators, oxygen cylinders, and liquid oxygen systems, including innovations in portability, oxygen generation, and automatic titration technologies (2004-2025).

Type of contents		Records, n
**Source of information**		
	Medicine	87
	Engineering	63
	Military	10
	Energy	5
	Space exploration	1
**Device**		
	Oxygen concentrator (including devices not on market)	156
	Oxygen cylinders	50
	Liquid oxygen	16
**New technology**		
	Downsize and portability enhancement	13
	Improving or optimizing the oxygen generation process	15
	Remote control (ie, automatic titration)	17
	Other	12

### Portable Oxygen Devices

**Figure 2:**
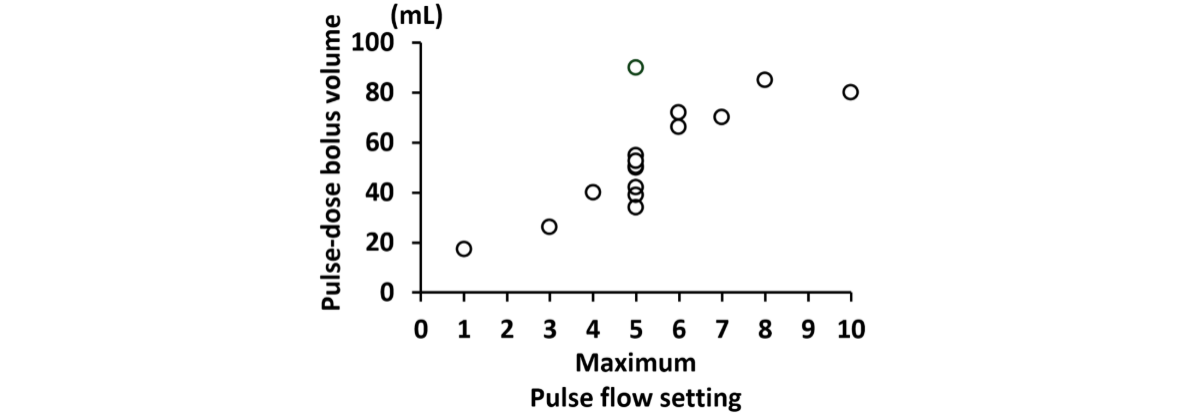
Scatter plot showing the relationship between maximum pulse flow setting and pulse-dose bolus volume (mL) in portable oxygen concentrators identified in this scoping review of ambulatory oxygen therapy devices. Data were extracted from publicly available scientific and gray literature published between 2004 and 2025. Only products with publicly available data on pulse-dose bolus volume at 20 breaths per minute at the maximum pulse flow setting are shown.

**Figure 3:**
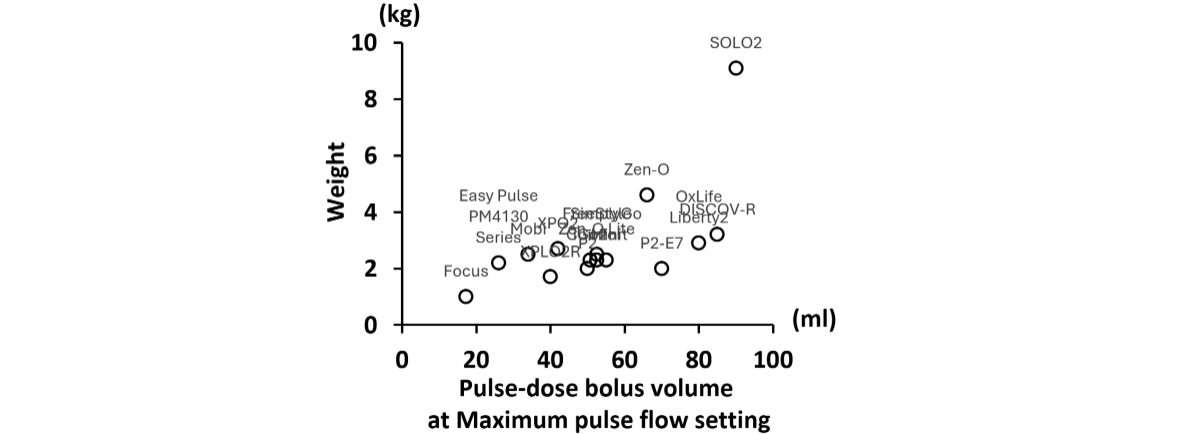
Scatter plot showing the relationship between product weight and pulse-dose bolus volume (mL) at the maximum pulse flow setting in portable oxygen concentrators identified in this scoping review of ambulatory oxygen therapy devices. Data were extracted from Lpublicly available scientific and gray literature published between 2004 and 2025. Only products with publicly available data on pulse-dose bolus volume at 20 breaths per minute at the maximum pulse flow setting are shown.

**Figure 4:**
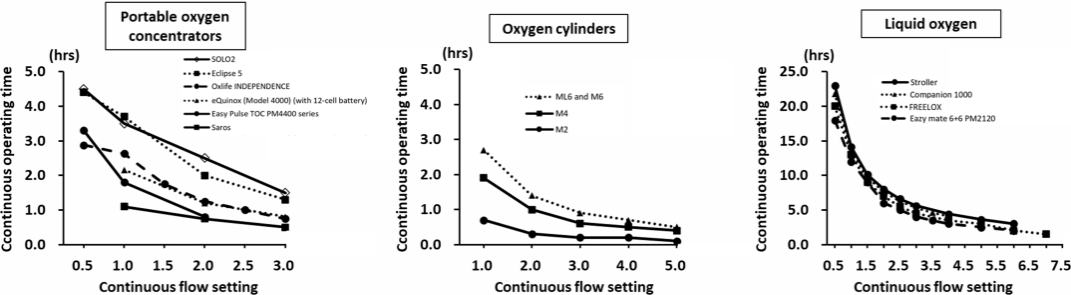
Continuous operating time at different continuous flow settings for (A) portable oxygen concentrators, (B) oxygen cylinders, and (C) liquid oxygen devices identified in this scoping review of ambulatory oxygen therapy devices. Data were extracted from publicly available product manuals and literature published between 2004 and 2025. Only products with publicly available data on continuous operating time for each continuous flow setting are shown.

We identified 33 different POCs in this study, of which 29 were on the market, 1 had discontinued production, and 3 were prototypes under development. The summary of identified POCs is shown in Table 4. The weight of the POCs products ranged from 1.0 to 9.1 kg, and size ranged from 15.7 × 11.7 × 6.1 cm (756 cm^3^) to 51.3 × 27.7 × 20.3 cm (28,847 cm^3^). Oxygen delivery capacity varied widely, with maximum flow rate in the continuous flow setting ranging from 2.0 to 6.0 L/min and maximum pulse-dose bolus volume of pulse flow setting ranging from 17.3 to 90 mL of oxygen. The pulse-dose bolus volume varies widely across products, even within the same pulse flow setting (Figure 2). A trade-off between portability and oxygen delivery was shown in the identified POCs (Figure 3, Table S3 in Multimedia Appendix 4). Smaller POC products tended to be transported using bags, while larger POC products were transported using a dedicated cart (Table S3 in Multimedia Appendix 4). Smaller POC products also tended to have only pulse flow settings. The most recent POCs were the OxLife Liberty2 (25.4 × 22.9 × 8.9 cm), released in 2024, and the DISCOV-R (23.6 × 10.4 × 25.2 cm), released in 2023, which were capable of delivering oxygen in the continuous flow setting. This contrasts with earlier POCs of the same size (eg, Inogen Rove 6, 18.3 × 8.3 × 20.5 cm with single battery), which only had a pulse flow setting (Table S3 in Multimedia Appendix 4). The mean maximum continuous operating time in pulse flow setting was 3.8 hours, with a range of 1.3 to 6.3 hours, depending on flow settings and battery option (Table 4 and Figure 4). The maximum operating time tended to be shortened by half in the continuous flow setting compared to the same flow rate in the pulse flow setting in terms of equivalent volume (Table 4 and Table S3 in Multimedia Appendix 4). There were also POC products, such as the Simply Go mini, which extended the continuous operating time from 2 hours 40 minutes to 5 hours with an additional battery (Table S3 in Multimedia Appendix 4).

Pressure swing adsorption (PSA), vacuum pressure swing adsorption (VPSA), and vacuum pressure cycle (VPC) were the technologies used for oxygen generation (Table 4, Table S4 in Multimedia Appendix 4). Zeolites were identified as the adsorbent used in POCs. Air is composed of approximately 80% nitrogen, and PSA is a method to generate high concentrations of oxygen by selectively adsorbing nitrogen from air. In PSA, air pressurized by a compressor is sent through an adsorption column with zeolite to adsorb nitrogen and increase oxygen concentration. Then, the pressure is reduced to release the nitrogen. This adsorption and desorption cycle process is alternated in the multiple adsorption columns to continuously produce highly concentrated oxygen [24]. VPSA and VPC are adsorption methods that use a vacuum pump in combination with or instead of a compressor to reduce the pressure in the column [25]. We identified 2 POC prototypes and 1 POC product that were under development. The 4-SLPM prototype developed by TDA Research (Wheat Ridge, United States) weighed approximately 7.8 kg and measured 28 × 25 × 18 cm (Table S3 in Multimedia Appendix 4). The 4-SLPM has a maximum oxygen flow rate of 4 L/min in continuous flow setting, greater than most POCs on the market of comparable weight. The 4-SLPM used high lithium-exchanged X (LiLSX) as the adsorbent. The other PSA-based prototype, co-developed by NASA and Chart Industries (Ball Ground, United States), weighed less than 8.2 kg with a size of 35.6 × 30.5 × 20.3 cm and was capable of delivering up to 6 L/min in the continuous flow setting. There was no available information on commercially available POC innovations, such as remote control of flow settings in POCs. A POC product under development named JUNO (Roam Technologies Pty Ltd, Carlton NSW, Australia) was identified. According to the company’s product information, JUNO is an ultraportable, tankless oxygen system currently under clinical development. Despite being designed to be carried with one hand, it has a continuous flow setting ranging from 1-3 L/min with a concentration of 91% [26]. Although the measurement conditions and detailed specification are not publicly disclosed (Table S3 in Multimedia Appendix 4), the recent patent on downsizing of POCs by Roam Technologies Pty Ltd, has been identified. The patent adopts a miniaturized PSA architecture with a compact arrangement of the adsorbent layer and internal gas pathway structure to shorten flow paths and minimize dead space [27].

Oxygen cylinders and LOX are summarized in Tables 5 and 6. Table 5 and Tables S3, S4, and S5 (shown in Multimedia Appendix 4) are also provided as xlsx files in Multimedia Appendix 5. The weight of cylinders ranged from 0.3-6.5 kg, and the size ranged from 14.9 × 6.4 cm to 86.5 × 10.2 cm. The maximum continuous operating time varied from 0.3 to 5.0 hours at 2 L/min in the continuous flow setting. The continuous operating time per continuous flow setting was comparable to the POC. (Figure 4). Smaller cylinders were transported using a shoulder bag, whereas larger cylinders required trolleys or hand-drawn carts for transport. Conventional cylinders were generally filled at approximately 150 bar, whereas the Ultra Lightweight Cylinder Oxygen System (IOSVR) uses a high-pressure filling of 300 bar. This allows nearly double the amount of oxygen to be filled compared with a comparably sized type M7 cylinder (Table 6). IOSVR is made possible by the high-strength aluminum alloy (L7X), which is reinforced with carbon fiber wrapping technology [28]. LOX products weighed from 1.6-3.7 kg (filled) and had gaseous oxygen capacities ranging from 275.0-1058 L (Table 6). Many oxygen cylinders require a pressure regulator, which is attached to the cylinder’s top and works like a tap, allowing the safe adjustment of oxygen flow rate provided, in L/min [4]. In addition, some regulators support a pulse-dose delivery mode, which can extend the operating duration compared with continuous flow [4,29].

**Table 4:** Summary of identified portable oxygen concentrators (n=33) included in this scoping review of ambulatory oxygen therapy devices. Extracted characteristics include device weight and dimensions, flow settings, oxygen output, operating noise, trigger sensitivity, oxygen delivery technology, regulatory approval, and market availability based on records published between 2004 and 2025.

Variables			Statistic		Notes
Device volume (cm^3^), mean (range)			8342.4 (755.9-28,846.5)		n=32
Weight (kg), mean (range)			3.8 (1.0-9.1)		n=16 (including battery); n=16 (unclear included)
**Transport method, n**			15		n=30; some devices fall into multiple categories
	Carrying bag	2			
	Carry case	2			
	Backpack	6			
	Cart	3			
	Shoulder strap	2			
	Waist	1			
	Carry-on baggage	3			
**PF^a^ and CF^b^ setting, n**					
	Only PF	19		N/A^c^	
	Both CF and PF	11		N/A	
	Not reported	2		N/A	
Maximum number of PF setting, mean (range)			5 (1-10)		n=29
Maximum flow rates in CF setting (L/min), mean (range)			2.9 (2.0-6.0)		n=12 (only devices with CF settings)
Maximum pulse-dose bolus in PF setting (mL), mean (range)			53.7 (17.3-90.0)		n=15 (Only those at 20 bpm)
Maximum continuous operating time in PF setting (h)^d^, mean (range)			3.8 (1.3-6.3)		n=25; depends on settings or respiratory rate
Maximum continuous operating time in CF setting (h)^d^, mean (range)			1.9 (0.6-4.5)		n=9; depends on settings or respiratory rate
Concentration of supplied oxygen (%)^d^, mean (range)			90.4 (82.0-96.0)		n=29; depends on settings or respiratory rate
Operating noise (dBA^e^)^d^, mean (range)			44.3 (35.0-59.0)		n=28; depends on settings or respiratory rate
Maximum battery recharge time (h), mean (range)			4.4 (1.5-12.8)		n=27
Trigger sensitivity (cm H_2_O), mean (range)			–0.24 (–0.50 to –0.05)		n=23
**Nature of the sorbent, n**					
	Molecular sieve	14		N/A	
	Zeolite	3		N/A	
	High lithium-exchanged X	1		N/A	
	Not reported	14		N/A	
**Technology used, n**					
	Pressure swing adsorption	7		N/A	
	Vacuum pressure swing adsorption	1		N/A	
	Vacuum pressure cycle	1		N/A	
	Not reported	23		N/A	
**Market availability, n**					
	On the market	29		N/A	
	Discontinued	1		N/A	
	Prototype	2		N/A	
**FDA^f^, n**					
	Approved	19		N/A	
	Not reported	13		N/A	
**FAA^g^, n**					
	Approved	28		N/A	
	Not reported	4		N/A	

^a^PF: pulse flow.

^b^CF: continuous flow.

^c^N/A: not applicable.

^d^JUNO (Roam Technologies Pty Ltd, Carlton NSW, Australia) was excluded from this analysis due to a significant lack of information.

^e^dBA: A-weighted decibels.

^f^FDA: Food and Drug Administration.

^g^FAA: Federal Aviation Administration.

**Table 5:** Summary of identified oxygen cylinders included in this scoping review of ambulatory oxygen therapy devices. Data were extracted from patents, manuals, and technological reports published between 2004 and 2025 and include cylinder size, weight, oxygen capacity, nominal pressure, maximum operating time, and transport methods (1 bar=100 kPa).

Type	Size (cm)	Weight (kg)	Content (L)	Nominal pressure (bar)	Maximum continuous operating time	Transport method
M2	14.9 × 6.4	0.34	45	153^a^	0.3 h at 2 L/min^a^	Not reported
M4	22.1 × 8.1	1.0	113	153^a^	1 h at 2 L/min^a^	Not reported
ML6	20.0 × 11.1	1.3	170	139^a^	1.4 h at 2 L/min^a^	Shoulder bag
M6	30.0 × 8.2	1.3	170	153^a^	1.4 h at 2 L/min^b^	Shoulder bag
M7	23.1 × 11.1	1.5	198	139^a^	1.7 h at 2 L/min^a^	Shoulder bag
IOSVR	34.2 × 8.5	1.7	612	300^§^	8 h at 2 L/min with a standard conserver^c^	Handheld or bag
M9	27.7 × 11.1	1.7	255	139^a^	2.1 h at 2 L/min^a^	Shoulder bag
D	53.5 × 10.2	3.9	340	140^a^	3.6 h at 2 L/min^a^	Shoulder bag
CH/C	51.5 × 11.7	4.2	470	163 or 250^a^	Not reported	Medical trolleys or carry bags
E	86.5 × 10.2	6.5	680	137^a^	5 h at 2 L/min^c^	Hand-drawn cylinder cart

^a^Data obtained from nonmanufacturer websites.

^b^Data reported in the technical reports.

^c^Data reported in scientific reports.

LOX had a longer continuous operating time compared to POCs and cylinders, with a mean maximum continuous operating time of approximately 7.4 hours even at 2 L/min of continuous flow setting (Figure 4). There was no available information on the pulse-dose bolus volume of pulse flow setting in LOX.

**Table 6:** Summary of identified liquid oxygen systems included in this scoping review of ambulatory oxygen therapy devices. Data were extracted from manuals, patents, and technical reports published between 2004 and 2025 and include weight, oxygen capacity, flow settings, maximum operating duration, and regulatory approval.

Variables					Statistic		Notes
Device volume (cm³), mean (range)					7889.2 (3650.2-13879.1)		n=6
Weight (kg), mean (range)					3.00 (1.6-3.7)		n=6
**Transport method, n**							Some devices fall into multiple categories
			Backpack		3		
			Cart		3		
			Carry bag		1		
			Belt pack		3		
			Handle		1		
Gaseous oxygen capacity (L), mean (range)					699.4 (275.0-1058.0)		N/A^a^
**PF^b^ or CF^c^ setting, n**							
	Only PF			3		N/A	
	Both CF and PF			3		N/A	
Maximum number of PF setting (n), mean (range)					5 (4-6)		n=3
Maximum flow rates in CF setting (L/min), mean (range)					4.3 (0.8-7)		n=6
Maximum continuous operating time in CF setting (h), mean (range)					7.4 (6.0-9.0)		n=5 (only those at 2 L/min of CF setting)
**FDA^d^, n**							
		Approved			5		N/A
		Not approved			1		N/A

^a^N/A: not applicable.

^b^PF, pulse flow.

^c^CF, continuous flow.

^d^FDA, Food and Drug Administration.

### Developments and New Technologies

#### Improving Portability

A patent was published in 2022 regarding the use of the vacuum swing adsorption method instead of the conventional PSA method in POCs to generate oxygen more efficiently [30]. By replacing the compressor in the PSA method with a fan or other air-moving device, air at atmospheric pressure instead of high pressure is passed through the zeolite adsorbent to adsorb nitrogen, and then the pressure is reduced to a vacuum level, which improves the efficiency of nitrogen desorption, resulting in improved oxygen generation rates [25]. This low-pressure operation makes it possible to use a small vacuum pump without a compressor in PSA, thus reducing the weight of the device and power consumption. Specifically, the POC in this patent had a device weight of 1.3 kg and a maximum oxygen flow rate of 1.5 L/min in the continuous flow setting. In addition, the POC in this patent uses LiLSX, which has recently been developed with improved selectivity and adsorption capacity for nitrogen [25,30,31]. A technology has been incorporated to maximize the efficiency of oxygen generation by monitoring the work of the zeolite adsorbent and the flow rate between the inlet and outlet of the adsorption column in real time to accurately detect the point when the adsorbent is saturated with nitrogen during the adsorption process (breakthrough point) and switch the cycle just before the saturated nitrogen is mixed with the concentrated oxygen. This allows oxygen to be separated without waste and maintains high oxygen purity while reducing the size of the device [30]. Another patent was published in 2023 for the design of a nasal-wearable POC entitled “Device and Method of Generating an Enriched Gas Within a Nasal Vestibule” [32]. However, few details were provided on the technical solutions used to achieve this small POC.

#### Improving or Optimizing the Oxygen Generation Process

A 2-bed rapid pressure swing adsorption (RPSA) system as an oxygen generation process was described in a scientific paper in 2021 [33]. The adsorption and desorption cycles performed in multiple adsorption columns in a conventional PSA system take a certain amount of time, which limits the amount of oxygen delivery. In this study, the cycle time was significantly reduced while maintaining a high oxygen concentration by optimizing the adsorbent and parameters such as pressure and cycle time related to adsorption. As a result, high oxygen productivity was observed despite the much lower ratio of adsorbent volume required (bed size factor) [33]. Other scientific papers have shown that sensitivity analysis modelling of PSA systems has led to optimization of operating conditions such as adsorption pressure, cycle time and adsorption bed size, leading to their applicability as oxygen supply infrastructure in small medical facilities and in high-humidity environments [34,35].

#### Remote Control

A scientific report for SpO_2_-targeted automatic titration of oxygen delivery during activities of daily living (ADL) testing based on the user’s SpO_2_ was identified [36]. This double-blinded randomized crossover trial evaluated the effect of SpO_2_-targeted automatic titration during ADL testing in 31 patients with chronic obstructive pulmonary disease (COPD) on long-term oxygen therapy. In this trial, an oxygen cylinder was connected to the closed-loop device (O2matic, Herlev, Denmark), and this device was placed on a rollator during ADL testing. In the active arm, automatic titration of oxygen flow rate (0-8 L/min) was set to aim at keeping an SpO_2_ target range of 90%-94% and those adjustments were done every second based on the average SpO_2_ for the last 15 seconds. In the control arm, the flow rate was kept fixed according to each patient’s medical prescription. As a result, automatic titration increased flow rates by triple and reduced ADL completion time, improved dyspnea, and reduced the number of events of severe desaturation compared to the control arm. Another technology for SpO_2_-targeted adjustment of oxygen delivery based on the user’s SpO_2_ was identified [37]. An “intelligent oxygen concentrator” for patients with COPD has been developed as a system that measures physical activity levels and automatically adjusts oxygen delivery according to machine learning (eg, decision tree, probabilistic neuronal network, logistic regression) [38]. This machine learning model was trained using patient activity data to discriminate the patient’s activity level (sedentary, light activity, and moderate activity). In a pilot study of 5 patients with COPD with long-term oxygen therapy, machine learning was used to automatically adjust the flow setting of pulse flow to match each patient’s physical activity level. As a result, the cumulative time of SpO_2_ below 90% when walking the circuit course was significantly reduced compared to manual changes [39].

#### Other Developments and New Technologies

A patent was identified for a new oxygen concentration control technique that delivers oxygen at low to moderate concentrations (eg, 30%-50%) by adjusting the pressure during the PSA cycle [40]. It was mentioned that the importance of this low-concentration oxygen delivery in clinical practice is unclear, but this patent could lead to reduced energy consumption of the POC, which has implications for extending battery life. In addition, a number of technologies for oxygen delivery control systems using sensors have been reported in recent years. One patent described sensors that use light to detect the timing of inhalation and valves that open and close electromagnetically at that timing to inject oxygen for a very short time (microbursts) [41]. This allows oxygen to be delivered with less waste of gas. In addition, a new delivery method was reported in which oxygen is generated and stored in advance and released in immediate response to inhalation [42].

### Cost

There were no available records of the cost of POCs officially disclosed by POC companies. Two identified scientific papers referred to POC prices of approximately US $2000 and US $3000 as retail costs to end users [43,44]. There were also no records of specific costs for LOX and oxygen cylinders. However, an identified article reported that LOX was approximately 4 times as expensive as standard oxygen therapy using POCs or portable cylinders [45]. Another identified article indicated that LOX systems entail higher service costs due to the need for regular home refills [46].

### Gap Map

**Figure 5:**
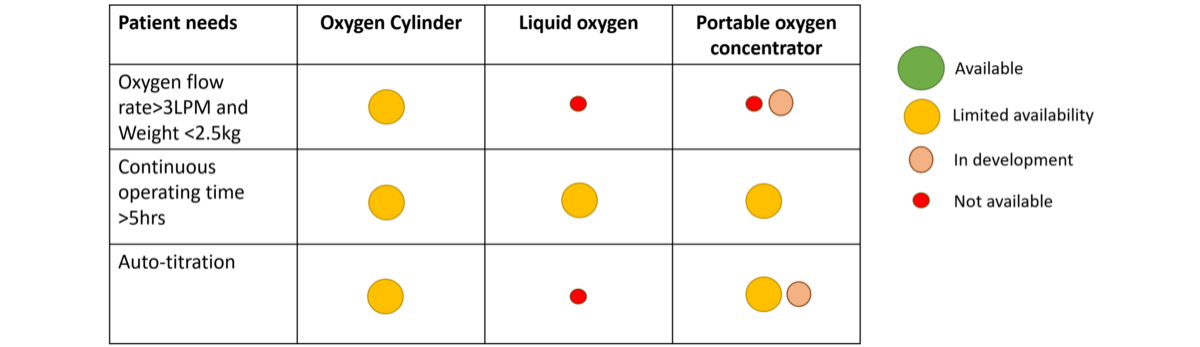
Gap map of patient needs and device availability in ambulatory oxygen therapy. The figure illustrates the extent to which current devices address 3 major patient needs: green indicates needs are met (“available”), yellow indicates limited devices meet patient needs (“limited availability”), orange indicates no current devices but technologies are under development (“in development”), and red indicates no available devices and no technologies under development (“not available”).

The gap map (Figure 5) showed that only a limited range of oxygen cylinders met patient needs for high flow rates (>3 L/min) and light weight (<2.5 kg). Although current POCs did not meet this need, a one-hand carry device capable of delivering 3 L/min was under clinical development. In all device types, few products met patient needs for continuous operating time (>5-6 hours). For auto-titration, SpO_2_-targeted automatic titration was available for POCs and cylinders as an interface, and the development of automatic titration capabilities embedded into the device itself has been reported only in POCs.

## Discussion

### Overview

This scoping review aimed to identify the range of portable oxygen devices, their oxygen generation technologies, performance characteristics, innovations in the design of ambulatory oxygen therapy devices, and cost. We identified 33 POCs, 10 oxygen cylinders, and 6 portable LOX systems. There was a trade-off between the portability (weight) and oxygen delivery capacity of POCs. The mean maximum continuous operating time of POCs was 3.8 hours. PSA with zeolite was the most common oxygen generation technology in POCs. Two POC prototypes were identified that had better oxygen delivery capacity despite being the same size as POCs on the market. LOX was the most portable and had the best continuous operating time among the devices. In terms of development and innovation, the downsizing of POCs using nanozeolite as the adsorbent, improving flow rate using RPSA and SpO_2_-targeted automated oxygen titration based on to patient’s SpO_2_ and physical activity were identified. Costs were rarely disclosed by manufacturers, and published data indicated that LOX is substantially more expensive than POCs or cylinders.

This study showed that high-flow oxygen delivery of 3 L/min in the continuous flow setting in POCs is only possible from commercially available POCs weighing more than 7.2 kg (eg eQuinox, Table S3 in Multimedia Appendix 4), which need to be transported on a cart. This may be a burden for patients requiring high flow rates. Furthermore, the short continuous operating time of the POCs identified in this study may also limit daily activities and community engagement. Patients have previously identified a desirable operating time for portable oxygen devices of 5-6 hours [9]. In the identified POC products, the mean maximum continuous operating time was up to 3.8 hours in the pulse flow setting. As higher flow rates shorten the continuous operating time, it is likely that fewer devices will meet patients’ needs. Although an additional battery can be used to double the operating time (eg, Simply Go), carrying the additional battery may be a burden for the patient. Furthermore, it should be noted that the numerical setting of a pulse flow does not correspond to the continuous flow rate and that the bolus volume varies between products even within the same pulse flow rate setting [47].

Among the devices, LOX had the best portability and continuous operating time, but the complicated maintenance was an obstacle. Previous studies have recognized LOX as a device with both better portability and continuous operating time [8], and have reported that LOX use is associated with better quality of life and physical activity compared to POCs and cylinders [48,49]. On the other hand, LOX requires refilling of liquid oxygen by a supplier at least 2-3 times a month, which imposes a high cost on the supplier and user [4,45]. As a result, the number of suppliers servicing LOX has been declining in the United States [8].

It was suggested that the trade-off between portability and oxygen delivery capacity is improving based on the latest products and prototypes identified. However, even in the latest POC products (DISCOV-R released in 2023 and OxLife Liberty2 released in 2024), the maximum oxygen flow rate of the continuous flow setting was still 2 L/min (Table S3 in Multimedia Appendix 4). JUNO, which is designed to be carried with one hand, is under clinical development and has a continuous flow setting ranging from 1-3 L/min with a concentration of 91% [26], and may be an innovative device, as the PSA Prototype was published in 2015 and the 4-SLPM in 2018 [50,51]. In terms of technological development, the nanozeolite (LilSX) had been of interest because of its use in 4-SLPM prototypes, patents, and basic research [30,52]. Although nanozeolites are characterized by higher nitrogen adsorption capacity, they are more expensive than conventional zeolites and have challenges such as long-term structural stability and degradation [53,54]. Although RPSA systems can improve oxygen productivity by shortening adsorption–desorption cycles, their application to POCs remains challenging because no clinical application studies have yet been reported [52]. Several recent clinical trials and patents described systems capable of adjusting flow rates based on the user’s SpO_2_ and physical activity. In a clinical trial with a small sample size, automated titration improved functional capacity in ADL, dyspnea, and the number of severe hypoxemia events [37]. However, these technologies have barriers, including sensor reliability or inaccuracy even during motion, requiring further development and clinical trials [37,55]. Identified patents in this study should be carefully considered because approximately half of the patents in general are not commercialized and launched on the market [56].

### Limitations

Limitations of this scoping review include the following. First, it is difficult to compare performance such as pulse-dose bolus volumes and continuous operating time between POCs because they depend on the number of breaths and device settings [57]. Second, this scoping review included only information published in English, which may have underestimated information on devices developed and used in non–English-speaking countries. Third, the pulse-dose bolus volume of POC at maximum pulse flow setting (as much as possible at a respiratory rate of 20 breaths per minute) extracted in this study does not exactly indicate the product’s oxygen delivery capacity. POC products vary in their algorithms for converting pulse-bolus volume and flow rate in response to changes in respiratory rate [57]. Fourth, available evidence regarding product performance relied almost on manufacturer-reported specifications, and no available evidence on device lifespan, failure rates, or long-term durability was available. There was no information available on the costs of POC products disclosed by manufacturers in this study. Lastly, the geographical distribution of the searched literature was biased toward the United States, as some gray literature was retrieved using domains such as “.gov,” “.mil,” and “.org” in Google Advanced Search.

### Conclusions

This scoping review is the first study to integrate medical, engineering, and gray literature evidence on ambulatory oxygen devices and map current ambulatory oxygen devices and their development status. Although prior literature has narratively described products and technologies for ambulatory oxygen therapy, no previous research has systematically investigated these products and new technologies. We identified 33 POCs, 10 oxygen cylinders, and 6 LOX systems. We showed the performance limitations of current devices and gaps in technological development in ambulatory oxygen therapy and suggested directions for future research and development. Specifically, POCs available to consumers may not meet patients’ needs in terms of oxygen flow rate, portability, and operating time. LOX offered the best performance in terms of operating time and portability among the devices but is restricted by high costs and declining availability. Although POCs are the most widely developed devices, technological innovation to achieve high oxygen flow rates, better portability, and longer continuous operating time has been limited since the POC prototype published in 2015. Collaboration among device developers, researchers, health professionals, and patients is urgently required to develop new lightweight devices with greater oxygen delivery capacity. It is essential to incorporate consumer input from the early stages of design and testing to ensure that future portable oxygen devices meet patients’ needs.

## Appendix

Multimedia Appendix 1 PRISMA-ScR-Fillable-Checklist_revised.

Multimedia Appendix 2 PRISMA 2020 for Abstracts Checklist.

Multimedia Appendix 3 Preferred Reporting Items for Systematic reviews and Meta-Analyses extension for Scoping Reviews (PRISMA-ScR) Checklist.

Multimedia Appendix 4 Additional material.

Multimedia Appendix 5 The performance characteristics of each identified products.

## Abbreviations

ADL: activities of daily living
COPD: chronic obstructive pulmonary disease
IOSVR: Ultra Lightweight Cylinder Oxygen System
JBI: Joanna Briggs Institute
LiLSX: high lithium-exchanged X
LOX: liquid oxygen
MeSH: Medical Subject Headings
POC: portable oxygen concentrator
PRISMA-S: Preferred Reporting Items for Systematic Reviews and Meta-Analyses Search extension
PRISMA-ScR: Preferred Reporting Items for Systematic reviews and Meta-Analyses extension for Scoping Reviews
PSA: pressure swing adsorption
RPSA: rapid pressure swing adsorption
SpO_2_: pulse oximeter oxygen saturation
VPC: vacuum pressure cycle
VPSA: vacuum pressure swing adsorption
WIPO: World Intellectual Property Organization

## Data Availability

The datasets generated or analyzed during this study are available in the Open Science Framework Storage from the corresponding author on reasonable request.

## References

[1] Jacobs SS, Krishnan JA, Lederer DJ, Ghazipura M, Hossain T, Tan AM (2020). Home oxygen therapy for adults with chronic lung disease: an official American Thoracic Society clinical practice guideline. Am J Respir Crit Care Med.

[2] Lacasse Y, Lecours R, Pelletier C, Bégin R, Maltais F (2005). Randomised trial of ambulatory oxygen in oxygen-dependent COPD. Eur Respir J.

[3] Jarosch I, Gloeckl R, Damm E, Schwedhelm A, Buhrow D, Jerrentrup A (2017). Short-term effects of supplemental oxygen on 6-min walk test outcomes in patients with COPD: a randomized, placebo-controlled, single-blind, crossover trial. Chest.

[4] Hardavella G, Karampinis I, Frille A, Sreter K, Rousalova I (2019). Oxygen devices and delivery systems. Breathe (Sheff).

[5] Chen J, Katz I, Pichelin M, Zhu K, Caillibotte G, Noga M (2017). Comparison of pulsed versus continuous oxygen delivery using realistic adult nasal airway replicas. COPD.

[6] Melani AS, Sestini P, Rottoli P (2018). Home oxygen therapy: re-thinking the role of devices. Expert Rev Clin Pharmacol.

[7] Tikellis G, Hoffman M, Mellerick C, Burge AT, Holland AE (2023). Barriers to and facilitators of the use of oxygen therapy in people living with an interstitial lung disease: a systematic review of qualitative evidence. Eur Respir Rev.

[8] Jacobs SS, Lederer DJ, Garvey CM, Hernandez C, Lindell KO, McLaughlin S (2018). Optimizing home oxygen therapy: an official American Thoracic Society workshop report. Ann Am Thorac Soc.

[9] Jacobs SS, Lindell KO, Collins EG, Garvey CM, Hernandez C, McLaughlin S (2018). Patient perceptions of the adequacy of supplemental oxygen therapy: results of the American Thoracic Society Nursing Assembly Oxygen Working Group Survey. Ann Am Thorac Soc.

[10] Levac D, Colquhoun H, O'Brien KK (2010). Scoping studies: advancing the methodology. Implement Sci.

[11] Johannson KA, Pendharkar SR, Mathison K, Fell CD, Guenette JA, Kalluri M (2017). Supplemental oxygen in interstitial lung disease: an art in need of science. Ann Am Thorac Soc.

[12] Ejiofor SI, Bayliss S, Gassamma A, Turner AM (2016). Ambulatory oxygen for exercise-induced desaturation and dyspnea in chronic obstructive pulmonary disease (COPD): systematic review and meta-analysis. Chronic Obstr Pulm Dis.

[13] Albert RK, Au DH, Blackford AL, Casaburi R, Cooper J A, Long-Term Oxygen Treatment Trial Research Group (2016). A randomized trial of long-term oxygen for COPD with moderate desaturation. N Engl J Med.

[14] Sanchez-Morillo D, Muñoz-Zara P, Lara-Doña A, Leon-Jimenez A (2020). Automated home oxygen delivery for patients with COPD and respiratory failure: a new approach. Sensors (Basel).

[15] Tricco AC, Lillie E, Zarin W, O'Brien KK, Colquhoun H, Levac D (2018). PRISMA extension for scoping reviews (PRISMA-ScR): checklist and explanation. Ann Intern Med.

[16] (2024). JBI Manual for Evidence Synthesis.

[17] Landerdahl Stridsberg S, Richardson MX, Redekop K, Ehn M, Wamala Andersson S (2022). Gray literature in evaluating effectiveness in digital health and health and welfare technology: a source worth considering. J Med Internet Res.

[18] Godin K, Stapleton J, Kirkpatrick SI, Hanning RM, Leatherdale ST (2015). Applying systematic review search methods to the grey literature: a case study examining guidelines for school-based breakfast programs in Canada. Syst Rev.

[19] Rethlefsen ML, Kirtley S, Waffenschmidt S, Ayala A, Moher D, Page M (2021). PRISMA-S: an extension to the PRISMA Statement for reporting literature searches in systematic reviews. Syst Rev.

[20] covidence.

[21] WHO technical specifications for oxygen concentrators. World Health Organization.

[22] Peters MDJ, Godfrey CM, Khalil H, McInerney P, Parker D, Soares CB (2015). Guidance for conducting systematic scoping reviews. Int J Evid Based Healthc.

[23] Blakeman TC, Rodriquez D, Britton TJ, Johannigman JA, Petro MC, Branson RD (2016). Evaluation of oxygen concentrators and chemical oxygen generators at altitude and temperature extremes. Mil Med.

[24] Yadav VK, Choudhary N, Inwati GK, Rai A, Singh B, Solanki B (2023). Recent trends in the nanozeolites-based oxygen concentrators and their application in respiratory disorders. Front Med (Lausanne).

[25] Ackley MW (2019). Medical oxygen concentrators: a review of progress in air separation technology. Adsorption.

[26] Tankless Oxygen Live Life Without Limits.

[27] Shan-Shan WE, Sisi Z, Jay RF, Jeremy TK, Natalie EH, Gavin DM (2024). Oxygen concentrator. WIPO.

[28] Bruton A, Sinclair I, Arnold E, Hepples W, Kay F, Maisey G (2012). The design and development of a new light-weight portable oxygen system. J Med Devices.

[29] Palwai A, Skowronski M, Coreno A, Drummond C, McFadden ER (2010). Critical comparisons of the clinical performance of oxygen-conserving devices. Am J Respir Crit Care Med.

[30] Jagger TW, Van Brunt NP, Kivisto JA, Lonnes PB (2022). Removable cartridge for oxygen concentrator. WIPO.

[31] Arora A, Hasan MMF (2021). Flexible oxygen concentrators for medical applications. Sci Rep.

[32] Assani KD (2023). Device and method of generating an enriched gas within a nasal vestibule. WIPO.

[33] Qadir S, Li D, Gu Y, Yuan Z, Zhao Y, Wang S (2021). Experimental and numerical analysis on the enhanced separation performance of a medical oxygen concentrator through two-bed rapid pressure swing adsorption. Ind Eng Chem Res.

[34] Benkirane L, Samid A, Chafik T (2023). Small-scale medical oxygen production unit using PSA technology: modeling and sensitivity analysis. J Med Eng Technol.

[35] Prayoga GA, Husni E, Damar Jaya S (2023). Design of an embedded controller and optimal algorithm of PSA for a novel medical oxygen concentrator. Int J Electr Eng Inform.

[36] Kofod LM, Hansen EF, Brocki BC, Kristensen MT, Roberts NB, Westerdahl E (2025). Optimised oxygenation improves functional capacity during daily activities in patients with COPD on long‑term oxygen therapy: a randomised crossover trial. Thorax.

[37] Prayoga GA (2023). Design and implementation system of mobile oxygen concentrator and telemedicine for comprehensive treatment of SpO_2_. Int J Adv Technol Eng Explor.

[38] Lara-Doña A, Sánchez‑Morillo D, Pérez‑Morales M, Fernández‑Granero MÁ, León‑Jiménez A (2019). A prototype of intelligent portable oxygen concentrator for patients with COPD under oxygen therapy.

[39] Sanchez-Morillo D, Muñoz-Zara P, Lara-Doña A, Leon-Jimenez A (2020). Automated home oxygen delivery for patients with COPD and respiratory failure: a new approach. Sensors (Basel).

[40] Warren J (2022). Efficient enriched oxygen airflow systems and methods. Wearair Ventures Inc.

[41] James JRJ, Zuzana EM, inventor SHL (2022). Pulsed oxygen system and process. Seabeck Holdings LLC.

[42] Wang Q, Liu Y (2022). Integrated oxygen supply device. Telesair Inc.

[43] Mapel DW, Robinson SB, Lydick E (2008). A comparison of health-care costs in patients with chronic obstructive pulmonary disease using lightweight portable oxygen systems versus traditional compressed-oxygen systems. Respir Care.

[44] Casaburi R, Hess M, Porszasz J, Clark W, Diesem R, Tal-Singer R, Ferguson C (2023). Evaluation of over-the-counter portable oxygen concentrators utilizing a metabolic simulator. Respir Care.

[45] Law S (2005). Liquid Oxygen Therapy at Home.

[46] Dunne PJ (2009). The clinical impact of new long-term oxygen therapy technology. Respiratory care.

[47] Dunne PJ (2012). Long-term oxygen therapy (LTOT) revisited: in defense of non-delivery LTOT technology. Rev Port Pneumol.

[48] Andersson A, Ström K, Brodin H, Alton M, Boman G, Jakobsson P (1998). Domiciliary liquid oxygen versus concentrator treatment in chronic hypoxaemia: a cost-utility analysis. Eur Respir J.

[49] Su C, Lee C, Chen H, Feng L, Lin H, Chiang L (2014). Comparison of domiciliary oxygen using liquid oxygen and concentrator in northern Taiwan. J Formos Med Assoc.

[50] Alptekin G (2018). Low power medical oxygen concentrators for space missions.

[51] Gilkey KM, Olson SL (2015). Evaluation of the Oxygen Concentrator Prototypes: Pressure Swing Adsorption Prototype and Electrochemical Prototype.

[52] Qadir S, Li D, Gu Y, Yuan Z, Zhao Y, Wang S (2021). Experimental and numerical analysis on the enhanced separation performance of a medical oxygen concentrator through two-bed rapid pressure swing adsorption. Ind Eng Chem Res.

[53] Pan M, Omar H, Rohani S (2017). Application of nanosize zeolite molecular sieves for medical oxygen concentration. Nanomaterials (Basel).

[54] Yadav VK, Choudhary N, Inwati GK, Rai A, Singh B, Solanki B (2023). Recent trends in the nanozeolites-based oxygen concentrators and their application in respiratory disorders. Front Med (Lausanne).

[55] Mondal A, Dutta D, Chanda N, Mandal N, Mandal S (2024). RESPIPulse: machine learning assisted sensory device for pulsed mode delivery of oxygen bolus using surface electromyography (sEMG) signals. Sens Actuators A Phys.

[56] Svensson R (2020). The scientific output of a database on commercialized patents. IFN Working Paper No. 1349.

[57] Chatburn RL, Williams TJ (2010). Performance comparison of 4 portable oxygen concentrators. Respir Care.

