# Supplementary material for: Products, Performance, and Technological Development of Ambulatory Oxygen Therapy Devices: Scoping Review
Source: J Med Internet Res. 2026 Jan 27;28:e81077. doi: 10.2196/81077 (PMC12892029; doi:10.2196/81077)
Supplement: Multimedia Appendix 4 [file jmir_v28i1e81077_app4.docx]

Table S1 Search strings

MEDLINE(R) ALL (Ovid) search strategy

| 1 | ((medical adj3 oxygen) or (oxygen adj3 treat) or (deliver adj3 oxygen) or (Oxygen adj3 therapy) or (long term adj2 oxygen therapy) or LTOT or (home adj2 oxygen therapy) or (ambulat adj3 oxygen) or (oxygen adj3 exercise) or (Suppl adj3 oxygen) or (HOT adj5 home) or (domiciliary adj3 oxygen)).mp. |
| --- | --- |
| 2 | Oxygen Inhalation Therapy/ MeSH term |
| 3 | 1 or 2 |
| 4 | ((instrument adj3 oxygen) or (compress adj3 oxygen cylinder) or (Oxygen adj2 cylinder) or (concentrator adj2 oxygen) or (POC adj5 portable) or liquid oxygen or LOX or PLOT or (machine adj3 oxygen) or (Oxygen adj2 generator) or (Product adj3 oxygen)).mp. |
| 5 | (oxygen adj10 (device or cylinder or concentrator or instrument)).mp. |
| 6 | 4 or 5 |
| 7 | 3 and 6 |
| 8 | Equipment Design/ MeSH term |
| 9 | Equipment Failure Analysis/ MeSH term |
| 10 | (flow rate or flow meter or battery or manufacturer or operating or transporting or electricity or wireless or remote control or breakdown or noise or loud or Sound or accident or Out of order).mp. |
| 11 | (FDA or food drug administration or EMA or european medicines agency or FAA or federal aviation administration or air travel or airplane or aeroplane or aircraft).mp. |
| 12 | ((damage or performance or characteristic or quality) adj15 (equipment or device or machine or instrument or gadget or apparatus or appliance or hardware)).mp. |
| 13 | ((pulse or continuous) adj10 (mode or flow or set)).mp. |
| 14 | "Equipment and Supplies"/ MeSH term |
| 15 | ((size or weight or concentration or product) adj10 (equipment or device or machine or instrument or gadget or apparatus or appliance or hardware or accessory)).mp. |
| 16 | 8 or 9 or 10 or 11 or 12 or 13 or 14 or 15 |
| 17 | 7 and 16 |
| 18 | limit 17 to english language |
| 19 | limit 18 to yr="2004 -Current" |
| 20 | (Adsorb or zeolite or PSA or (sieve bed or molecular sieve or sieve type) or pressure swing adsorption).mp. |
| 21 | ((technology or mechanism or Develop or Preparator Adjust) adj15 (flow or function or performance or equipment or device or machine or instrument or gadget or apparatus or appliance or hardware)).mp. |
| 22 | (innovation or start-up or venture capital or pilot).mp. |
| 23 | 20 or 21 or 22 |
| 24 | 7 and 23 |
| 25 | limit 24 to english language |
| 26 | limit 25 to yr="2004 -Current" |
| 27 | Health Care Costs/ MeSH term |
| 28 | Health Services Research/ MeSH term |
| 29 | (Costs and Cost Analysis).mp. |
| 30 | Cost-Benefit Analysis/ MeSH term |
| 31 | (expense or cost or bill or charge or fees or expenditure or payment or spending or economic evaluation or economic factor or funds or financial or financing).mp. |
| 32 | 27 or 28 or 29 or 30 or 31 |
| 33 | 7 and 32 |
| 34 | limit 33 to english language |
| 35 | limit 34 to yr="2004 -Current" |

Embase (Ovid) search strategy

| 1 | ((medical adj3 oxygen) or (oxygen adj3 treat) or (deliver adj3 oxygen) or (Oxygen adj3 therapy) or (long term adj2 oxygen therapy) or LTOT or (home adj2 oxygen therapy) or (ambulat adj3 oxygen) or (oxygen adj3 exercise) or (Suppl adj3 oxygen) or (HOT adj5 home) or (domiciliary adj3 oxygen)).mp. |
| --- | --- |
| 2 | Oxygen Inhalation Therapy/ MeSH term |
| 3 | 1 or 2 |
| 4 | ((instrument adj3 oxygen) or (compress adj3 oxygen cylinder) or (Oxygen adj2 cylinder) or (concentrator adj2 oxygen) or (POC adj5 portable) or liquid oxygen or LOX or PLOT or (machine adj3 oxygen) or (Oxygen adj2 generator) or (Product adj3 oxygen)).mp. |
| 5 | (oxygen adj10 (device or cylinder or concentrator or instrument)).mp. |
| 6 | 4 or 5 |
| 7 | 3 and 6 |
| 8 | Equipment Design/ MeSH term |
| 9 | Equipment Failure Analysis/ MeSH term |
| 10 | (flow rate or flow meter or battery or manufacturer or operating or transporting or electricity or wireless or remote control or breakdown or noise or loud or Sound or accident or Out of order).mp. |
| 11 | (FDA or food drug administration or EMA or european medicines agency or FAA or federal aviation administration or air travel or airplane or aeroplane or aircraft).mp. |
| 12 | ((damage or performance or characteristic or quality) adj15 (equipment or device or machine or instrument or gadget or apparatus or appliance or hardware)).mp. |
| 13 | ((pulse or continuous) adj10 (mode or flow or set)).mp. |
| 14 | "Equipment and Supplies"/ MeSH term |
| 15 | ((size or weight or concentration or product) adj10 (equipment or device or machine or instrument or gadget or apparatus or appliance or hardware or accessory)).mp. |
| 16 | 8 or 9 or 10 or 11 or 12 or 13 or 14 or 15 |
| 17 | 7 and 16 |
| 18 | limit 17 to english language |
| 19 | limit 18 to yr="2004 -Current" |
| 20 | (Adsorb or zeolite or PSA or (sieve bed or molecular sieve or sieve type) or pressure swing adsorption).mp. |
| 21 | ((technology or mechanism or Develop or Preparator Adjust) adj15 (flow or function or performance or equipment or device or machine or instrument or gadget or apparatus or appliance or hardware)).mp. |
| 22 | (innovation or start-up or venture capital or pilot).mp. |
| 23 | 20 or 21 or 22 |
| 24 | 7 and 23 |
| 25 | limit 24 to english language |
| 26 | limit 25 to yr="2004 -Current" |
| 27 | Health Care Costs/ MeSH term |
| 28 | Health Services Research/ MeSH term |
| 29 | (Costs and Cost Analysis).mp. |
| 30 | Cost-Benefit Analysis/ MeSH term |
| 31 | (expense or cost or bill or chargeor fees or expenditure or payment or spending or economic evaluation or economic factor or funds or financial or financing).mp. |
| 32 | 27 or 28 or 29 or 30 or 31 |
| 33 | 7 and 32 |
| 34 | limit 33 to english language |
| 35 | limit 34 to yr="2004 -Current" |

Scopus search strategy

| 1 | TITLE-ABS-KEY ( ( treat OR therap OR medi ) AND ( ( deliver W/3 oxygen ) OR ( "long term" W/2 "oxygen therapy" ) OR ltot OR ( "home oxygen" W/2 therapy ) OR ( ambulat W/3 oxygen ) OR ( oxygen W/3 exercise ) OR ( suppl W/3 oxygen ) OR ( hot W/5 home ) OR ( domiciliary W/3 oxygen ) OR ( oxygen W/3 inhalation ) ) ) |
| --- | --- |
| 2 | TITLE-ABS-KEY ( ( instrument W/3 oxygen ) OR ( compress W/3 "oxygen cylinder" ) OR ( oxygen W/2 cylinder ) OR ( concentrator W/2 oxygen ) OR ( poc W/5 portable ) OR "liquid oxygen" OR lox OR plot OR ( machine W/3 oxygen ) OR ( oxygen W/2 generator ) OR ( product W/3 oxygen ) ) OR ( ALL ( oxygen W/10 ( device OR cylinder OR concentrator OR instrument ) ) ) |
| 3 | ( TITLE-ABS-KEY ( ( equipment W/3 design ) OR ( equipment W/3 failure ) ) ) OR ( TITLE-ABS-KEY ( "flow rate" OR "flow meter" OR battery OR manufacturer OR operating OR transporting OR electricity OR wireless OR "remote control" OR breakdown OR noise OR loud OR sound OR accident OR "out of order" ) ) OR ( TITLE-ABS-KEY ( fda OR "food drug administration" OR ema OR "european medicines agency" OR faa OR "federal aviation administration" OR "air travel" OR airplane OR aeroplane OR aircraft ) ) OR ( TITLE-ABS-KEY ( damage OR performance OR characteristic OR quality ) W/15 ( equipment OR device OR machine OR instrument OR gadget OR apparatus OR appliance OR hardware ) ) OR ( TITLE-ABS-KEY ( ( pulse OR continuous ) W/10 ( mode OR flow OR set ) ) ) |
| 4 | 1AND2AND3 |
|  | limit 4 to english language and yr="2004 -Current" |
| 5 | ( TITLE-ABS-KEY ( concentrat W/10 ( adsorb OR zeolite OR psa OR "sieve bed" OR "molecular sieve" OR "sieve type" OR "pressure swing adsorption" ) ) ) OR ( ( technology OR mechanism OR develop OR preparat OR adjust ) W/15 ( flow OR function OR performance OR equipment OR device OR machine OR instrument OR gadget OR apparatus OR appliance OR hardware ) ) OR ( innovation OR "start-up" OR venture AND capital OR pilot ) |
| 6 | 1AND2AND5 |
| 7 | limit 6 to english language and yr="2004 -Current" |
| 8 | TITLE-ABS-KEY ( ( "health care" W/3 cost ) OR ( "health service" W/3 research ) OR "cost analysis" OR ( "cost-benefit" W/3 analysis ) OR expense OR cost OR bill OR charge OR fees OR expenditure OR payment OR spending OR "economic evaluation" OR "economic factor" OR funds OR financial OR financing ) |
| 9 | 1AND2AND8 |
| 10 | limit 9 to english language and yr="2004 -Current" |
|  | Total of Scopus |

IEEE search strategy

| 1 | ((( deliver NEAR/3 oxygen ) OR ( "long term" NEAR/2 "oxygen therapy" ) OR ltot OR (home AND "oxygen therapy" ) OR ( ambulat NEAR/3 oxygen ) OR ( oxygen NEAR/3 exercise ) OR ( suppl NEAR/3 oxygen ) OR ( hot NEAR/5 home ) OR ( domiciliary NEAR/3 oxygen ) OR ( oxygen NEAR/3 inhalation )) AND (treat OR therap OR medi)) |
| --- | --- |
| 2 | ((((oxygen NEAR/10 ( device OR cylinder OR concentrator OR instrument))) OR (( instrument NEAR/3 oxygen ) OR ( compress NEAR/3 "oxygen cylinder" ) OR ( oxygen NEAR/2 cylinder ) OR ( concentrator NEAR/2 oxygen ) OR ( poc NEAR/5 portable ) OR "liquid oxygen" OR lox OR plot OR ( machine NEAR/3 oxygen ) OR ( oxygen NEAR/2 generator ) OR ( product NEAR/3 oxygen )))) |
| 3 | 1AND2 |
|  | limit 3 to yr="2004 -Current |

In the engineering database(IEEE), I searched for Concept A and Concept B only.

Search strategy for grey literature

| **National Technical Reports Library (Technical report)** | |
| --- | --- |
| “Oxygen therapy" "oxygen concentrator" "oxygen cylinder" "liquid oxygen" |  |
| **World Health Organization (white paper)** | |
| Oxygen |  |
| **Defense Technical Information Center (military medicine)** | |
| "Oxygen therapy" "oxygen concentrator" "oxygen cylinder" "liquid oxygen" |  |
| **International Health technology assessment (HTA) database (General Grey literature)** | |
| ((Oxygen therapy) OR (oxygen concentrator) OR (oxygen cylinder) OR (liquid oxygen)) |  |
| **Google advanced (General information including web site)** |  |
| All these words: oxygen therapy  Any of these words:"long-term" OR home OR ambulatory OR size OR "flow rate" OR "remote control" OR function OR characteristic OR failure OR sound OR FDA OR EMA OR FAA OR airplane OR zeolite OR psa OR "sieve bed" OR "pressure swing adsorption" OR technology OR develop OR cost OR expense  Site or domain: .com (company) |  |
| All these words: oxygen therapy  Any of these words:"long-term" OR home OR ambulatory OR size OR "flow rate" OR "remote control" OR function OR characteristic OR failure OR sound OR FDA OR EMA OR FAA OR airplane OR zeolite OR psa OR "sieve bed" OR "pressure swing adsorption" OR technology OR develop OR cost OR expense  Site or domain: .gov (goverment) |  |
| All these words: oxygen therapy  Any of these words:"long-term" OR home OR ambulatory OR size OR "flow rate" OR "remote control" OR function OR characteristic OR failure OR sound OR FDA OR EMA OR FAA OR airplane OR zeolite OR psa OR "sieve bed" OR "pressure swing adsorption" OR technology OR develop OR cost OR expense  Site or domain:.mil (the United States military) |  |
| **World Intellectual Property Organization;WIPO (Patent)** | |
| AD:([01.01.2023 TO 31.12.2024]) AND ((Oxygen NEAR3 therapy) or (long term NEAR2 oxygen therapy) or LTOT or (home NEAR2 oxygen therapy) or (ambulat NEAR3 oxygen) or (oxygen NEAR3 exercise) or (Suppl NEAR3 oxygen) or (HOT NEAR5 home) or (domiciliary NEAR3 oxygen) or (Oxygen NEAR3 inhalation)) AND ((instrument NEAR3 oxygen) or (compress NEAR3 oxygen cylinder) or (Oxygen NEAR2 cylinder) or (concentrator NEAR2 oxygen) or (POC NEAR5 portable) or (liquid oxygen) or (LOX) or (PLOT) or (machine NEAR3 oxygen) or (Oxygen NEAR2 generator) or (Product NEAR3 oxygen) or (oxygen NEAR10 device) or (oxygen NEAR10 cylinder) or (oxygen NEAR10 concentrator) or (oxygen NEAR10 instrument)) |  |

Table S2. Countries of origin of the record (n=166)

| Location of study, n (%) |  |  |
| --- | --- | --- |
| United States | 73 | 44.0% |
| India | 18 | 10.0% |
| Australia | 11 | 6.6% |
| United Kingdom | 8 | 4.8% |
| Canada | 8 | 4.8% |
| France | 5 | 3.0% |
| Germany | 5 | 3.0% |
| Japan | 5 | 3.0% |
| Spain | 6 | 3.6% |
| Switzerland | 3 | 1.8% |
| Brazil | 2 | 1.2% |
| Indonesia | 2 | 1.2% |
| Italy | 2 | 1.2% |
| Poland | 2 | 1.2% |
| WIPO | 2 | 1.2% |
| Belgium | 1 | 0.6% |
| China | 3 | 1.8% |
| Greece | 1 | 0.6% |
| Morocco | 1 | 0.6% |
| Norway | 1 | 0.6% |
| Pakistan | 1 | 0.6% |
| Sweden | 1 | 0.6% |
| Thailand | 2 | 1.2% |
| The Gambia | 1 | 0.6% |
| Turkey | 1 | 0.6% |
| Denmark | 1 | 0.6% |

Table S3. The performance characteristics of each identified portable oxygen concentrator

| Name | Manufacturer | Size | Weight | Transport  method | PF/CF  Setting | Setting of PF | Flow rates in CF | Pulse-dose bolus volume in PF | Maximum continuous operating time |
| --- | --- | --- | --- | --- | --- | --- | --- | --- | --- |
| Focus | AirSep | 15.7 (H) x 11.7(W) x 6.1 (D)cm | 1.0kg with battery | Carrying bag | Only PF | 1 | N/A | 17.25ml at 20bpm^**^ | 3h^*^ |
| Trooper | VBOX Inc (US) | 15.24 x 6.35 x 15.88 cm | 1.5kg with battery | Fanny pack or Purse or Soulder bag | Only PF | 1-5 | N/A | NR | 6h with both batteries^**^ |
| P2-S4 | Rhythm Healthcare | (Single Battery) 15.7 ｘ 8.6 x x17.0 cm (Double Battery)15.7 x 8.6 x 19.0 cm (Triple Battery)15.7 x 8.6 x 21 cm | 1.5kg with single battery/ 1.8kg with double battery/ 2.0kg with triple battery | Carrying bag | Only PF | 1-4 | N/A | 84ml^*^ | Up to 2.5h at Setting 1 of PF^**^ |
| XPLO_2_R | Belluscura | 18.5 x 7.4 x 19.3cm with 8-cell battery | 1.7kg with 8-cell battery | Backpack | Only PF | 1-4 | N/A | 40ml at setting 4 of PF/ 20bpm^*^ | 2h 25m with 8-cell battery at setting 5 of PF^*^ |
| P2-E7 | Rhythm Healthcare | 22.1 (L) x 8.5 (W) x 16.0 (D)cm | 2.0kg | Carrying bag | Only PF | 1-7 | N/A | 70ml at setting at 7 of PF/ 20bpm^*^ | 5.2h at setting 2 of PF^*^ |
| P2 | Rhythm Healthcare | 8.4 (W) x 22.1 (D) x 16.0 (H)cm | 2.0kg | Carrying bag | Only PF | 1-5 | N/A | 50ml at setting 5 of PF/ 20bpm^*^ | 1h 50m at setting 5 of PF^*^ |
| LifeChoice ActivOx Pro model | Inova Labs | 24.1(H) x 19.1 (W) x 7.9 (D)cm | 2.2kg | Carry case, back pack, shoulder strap, waist strap | Only PF | 1-3  LPMeq | N/A | Undisclosed^†^ | 6.25h at 3LPM LPMeq of PF^*^ |
| Easy Pulse PM4130 Series | Precision Medical | 21.6 (H) x 16.5 (W) x 11.4 (D)cm | 2.2kg (Shipping Weight: 14.91 kg) | Carrying bag | Only PF | 1-3 | N/A | 26.0 ml Setting 3 of PF/ at 20bpm^§^ | 4.0h at setting 2 of PF^§^ |
| ActiVox LifeChoice | Inova Labs | 23 x 20 x 11cm | 2.2kg with battery | Carry-on baggage | Only PF | 1-4  LPMeq | N/A | 30ml at 15bpm^§^ | 5h at 3LPM LPMeq of PF^§^ |
| Live Active 5 | Precision Medical | 21.4 (H) x 8.3(W) x 21.6 (D)cm | 2.3kg with single battery | Carry bag | Only PF | 1-5 | N/A | 1000 +/-15% mL/min at setting 5 of PF^*^ | 4.3h at setting 2 of PF^*^ |
| iGo2 | DeVilbiss Healthcare | 21.3 (H) x 8.9 (W) x 21.8 (L)cm | 2.3kg | Carrying Case(bag) | Only PF | 1-5 | N/A | 50.7ml at setting 5 of PF/ at 20bpm^*^ | A minimum of 3.5h at setting 2 of PF at 20 bpm^†^ |
| FreeStyle Comfort | Caire | 25.4 x 18.5 x 7.9 cm | 2.3kg with single battery/ 2.7kg with double battery | Carrying bag | Only PF | 1-5 | N/A | 52.5ml at setting 5 of PF/ at 20bpm^*^ | Up to 4h with single battery at setting 2 of PF/ Up to 8h with double battery at setting 2 of PF^*^ |
| SimplyGo Mini | Philips Respironics | 25.9 x 21.1 x 9.1cm | 2.3kg with battery | Accessory Bag or cart | Only PF | 1-5 | N/A | 55ml at setting 5 of PF/ at 20bpm^§^ | 4.5h^§^ |
| Zen-O Lite | GCE | 24.9 (W) x 9.7 (D) x 23.5 (H)cm | 2.5kg | Carrying bag | Only PF | 1-5 | N/A | 52.5ml at setting 5 of PF/ at 20bpm^§^ | 4.0h at setting 2 of PF^§^ |
| Mobi | ResMed | 20.2 (H) x 24.4 (W) x 11.4 (D)cm | 2.5kg | Carrying case with Shoulder strap | Only PF | 1-4 | N/A | 34.0ml at setting 4 of PF/ at 20bpm^§^ | 6.0h at setting 2 of PF^§^ |
| Hi-Sanso Portable α II | TEIJIN PHARMA | 24(W) x 11(D) x 21(H)cm | 2.5kg | NR | NR | NR | NR | NR | NR |
| XPO_2_ | Invacare Corporation | 25 x 18 x 10cm | 2.7kg with battery | Carrying bag | Only PF | 1-5 | N/A | 42.0ml at setting 5 of PF/ at 20bpm^§^ | 2.4h at setting 2 of PF^§^ |
| FreeStyle 5 | AirSep | 27.2 x 16.8 x 11.2cm | 2.8kg with battery | Backpack style or worn on the waist | Only PF | 1-5 | N/A | 43.75ml ± 10%^§^ | 1.25-4.25h^§^ |
| OxLife Liberty2 | O2 Concepts | 25.4 (H) x 22.9 (W) x 8.9 (D)cm | 2.9kg | Carrying bag | Both | 1-10 | 0.5-2LPM^*^ | 80ml at setting 10 /at 20bpm^*^ | 4h at setting 2 of PF^*^ |
| DISCOV-R | Belluscura | 23.6 (L) x 10.4 (W) x 25.2 (H)cm | 3.2kg with 8-cell battery | Carrying bag | Both | 1-8 | 0.5-2 LPM^*^ | 85ml at setting 8 of PF/ at 20bpm^*^ | Up to 4.5h with a battery^†^ |
| Inogen Rove 6 | Inogen Inc | 18.3 (L) x 8.3 (W) x 20.5 (H)cm with single battery | 3.3kg | Carrying bag | Only PF | 1-6 | N/A | 1.2LPM^†^ | 5h with standard battery/ 10h with extended battery^†^ |
| EverGo | Respironics | 21.6 (H) x 15.3 (W) x 30.5 (D)cm | 4.5kg with two batteries | Carrying Case with Detachable Handle and Shoulder Strap | Only PF | 1-6 | N/A | 12–70ml^§^ | 4.0h at setting 6 at 20bpm^§^ |
| Simply Go | Philips Respironics | 29.2 x 25.4 x 15.2 cm | 4.5kg | Accessory Bag | Both | 1-6 | 0.5-2.0 LPM^§^ | 72ml at setting 6 of PF^**^ | 2h 40m with single battery/ 5h with optional additional battery^§^ |
| Zen-O | GCE | 21.2 (W) x 16.8 (D) x 31.3 (H)cm | 4.6kg | Trolley | Both | 1-6 | 0.5-2.0 LPM^†^ | Max 66ml at setting 6 of PF/ at 20bpm^†^ | 0.75h at setting 2LPM of CF/ 4h at setting 2 of PF^†^ |
| Easy Pulse TOC PM4400 series | Precision Medical | 37.2(H) x 26.7(W) x 17.8(D)cm | 5.2kg | Bag or Cart | Both | 1-5 | 0.25-2.0 LPM^§^ | Up to39.0ml at setting 5 of PF^§^ | 0.80h at setting 2LPM of CF/ 3.0h at setting 2 of PF^§^ |
| Saros  (Designed for Military) | Caire | 68.7 (L) x 11.1 (Diameter) cm | 5.6kg with battery | Harness with shoulder strap | Both | 16, 32, 48, 64, 80, 96 ml | 1.0-3.0 LPM^§^ | 16-96 ml^§^ | 34.0 ± 2.2 m at setting 3LPM of CF^§^ |
| eQuinox (Model 4000) | Caire | 34.5 x 26.9 x 18.8 cm | 7.2kg with battery | Cart | Both | 1-9 | 0.5-3.0LPM^**^ | 192ml^**^ | 2.8h at setting 2 of PF/ 1.2 h at 2LPM of CF (with 12-cell battery)^*^ |
| Oxlife INDEPENDENCE | O2 Concepts | 51.3 × 27.7 × 20.3 cm | 7.6kg | Cart | Both | 0.51-6 | 0.5-3LPM^*^ | 96ml at setting 6 of PF^*^ | 1h 15min at 2LPM of CF/ 2h 53m at setting 2 of PF^*^ |
| Prototype 4 of the 4-SLPM † | TDA Research, Inc. (TDA) | 28 x 25 x 18cm | 7.8kg | NR | NR | NR | 1.0-4.0 LPM^§^ | NR | NR |
| Pressure Swing Adsorption (PSA) Prototype† | Chart Industries (under NSBRI grant) | approximately 35.6 (L) x 30.5 (W) x 20.3 (H) cm (not sure prototype of PSA or of POC) | 8.2kg | NR | Both | NR | 1-6 LPM^§^ | 16, 32, 48, 64, 80, 96 ml^§^ | NR |
| Eclipse 5 | Caire | 49.0 (H) x 31.2 (W) x 18.0 (D)cm | 8.3kg with battery | Cart | Both | 1-9 | 0.5-3.0 LPM^§^ | 192ml at setting 9 of PF^*^ | 1.3h at 3LPM of CF/ 1.7h at setting 9^*^ |
| SOLO_2_ | Invacare | 41.9 (H) x 27.9 (W) x 20.3 (D)cm | 9.1kg with battery | Cart | Both | 1-5 | 1-3 LPM^*^ | 90ml at setting 5 of PF/ at 20 bpm^*^ | 2.6h at 2LPM of CF^*^ |
| Juno Portable Oxygen Concentrator | Roam Technologies | NR | NR | NR | NR | 1-3 LPM^**^  (It is unclear whether it's in PF or CF) | | NR | NR |

**Note:** ^*^Data reported in the product manual; ^**^Data reported in the manufacturer’s official website; ^†^Data obtained from non-manufacturer websites; ^§^Data reported in the scientific reports.

**Abbreviations:** bpm, breaths per minute; CF, continuous flow; DC, direct current; LPM, liters per minute; LPM, liters per minute equivalent; NR, Not Reported; PF, pulse flow.

Table S4. The features and technologies used of each identified portable oxygen concentrator

| Device | Concentration of supplied oxygen | Operating noise | Battery recharge time | Trigger sensitivity | Nature of the sorbent in POC | Technology used | Release date | Market Availability | FDA approved | FAA approved |
| --- | --- | --- | --- | --- | --- | --- | --- | --- | --- | --- |
| Focus | 90 +5.5/ -3%^§^ | 45 dBA^*^ | 4h^*^ | Undisclosed | Molecular sieve | NR | 2018 | On the market | FDA approved | FAA approved |
| Trooper | 87 - 94% at all settings^*^ | NR | NR | ≤0.13 cm H_2_O | Zeolite | PSA | 2013 | On the market | FDA approved | FAA approved |
| P2-S4 | 87 - 96%^**^ | <45 dBA^†^ | 1/2/3h^†^ | ≤0.12 cm H_2_O^*^ | NR | NR | NR | On the market | NR | FAA approved |
| XPLO_2_R | 82 - 92% at all settings^*^ | <39 dB^*^ | ≤ 4h^†^ | ≤0.2 cm H2O^*^ | Molecular sieve | NR | NR | On the market | FDA approved | FAA approved |
| P2-E7 | 90 +6/ -3% at all settings^*^ | 35 dBA at setting 2^*^ | Not more than 4h^*^ | ≤0.12 cm H_2_O^*^ | Molecular sieve | NR | NR | On the market | FDA approved | FAA approved |
| P2 | 90 +6/ -3% at all settings^*^ | 38 dBA at setting 2^*^ | Not more than 4h^*^ | ≤0.12 cm H_2_O^*^ | NR | NR | NR | On the market | FDA approved | FAA approved |
| LifeChoice ActivOx Pro model | 90 ± 3%^*^ | 46 dB^†^ | Approximate 4.5h^*^ | Undisclosed^†^ | Molecular sieve | PSA | 2014 | On the market | FDA approved | FAA approved |
| Easy Pulse PM4130 Series | 90 +5/ -3%^§^ | 42 dBA at setting 2^§^ | NR | 0.50 cmH_2_O maximum^*^ | NR | NR | NR | On the market | NR | FAA approved |
| ActiVox LifeChoice | 90 ± 3%^*^ | 44 dBA^§^ | 4h^§^ | NR | NR | NR | NR | On the market | NR | FAA approved |
| Live Active 5 | 87% to 95.5^*^ | <40dBA^*^ | 6h^*^ | < -0.45 cmH2O^*^ | Molecular sieve | NR | NR | On the market | NR | FAA approved |
| iGo2 | 90 +4/ -3%^*^ | <37.5 dB^**^ | 3.5h^**^ | NR | Molecular sieve | NR | 2019 | On the market | FDA approved | FAA approved |
| FreeStyle Comfort | 90 +5.5/ -3%^*^ | 39.9dB at setting2^†^ | Single Battery: 3.5 h/ Double Battery: 6.0 h^*^ | > -0.5 cm H_2_O^*^ | Molecular sieve | PSA | NR | On the market | FDA approved | FAA approved |
| SimplyGo Mini | 90 +6/-3%^§^ | 43 dB at setting 2^§^ | 4h^§^ | ≤ 0.2 cm H2O^*^ | Molecular sieve | NR | NR | On the market | NR | FAA approved |
| Zen-O Lite | 90 +6/ -3%^§^ | 37 dBA at setting 2^§^ | 4.5h at setting 2^§^ | -0.12 cmH2O^*^ | Molecular sieve | NR | NR | On the market | NR | FAA approved |
| Mobi | 90 +6/ -3%^§^ | 53.5 dBA at setting 4^*^ | 4h^*^ | 0.140 cm H2O (Active mode), 0.101 cm H2O (Rest mode)^*^ | Molecular sieve | NR | 8 Jan 2019 | On the market | NR | FAA approved |
| Hi-Sanso Portable α II | NR | NR | NR | NR | NR | NR | NR | On the market | NR |  |
| XPO_2_ | 90 + 5.6/− 3^§^ | 44-46 dBA^§^ | 4h^§^ | 0.18 cmH2O^§^ | Molecular sieve | PSA | NR | On the market | FDA approved | FAA approved |
| FreeStyle 5 | 90 +5.5/ -3%^*^ | 41 dB at setting 2 48 dB at setting 3^§^ | 4-5h^§^ | -0.054 cm H2O^†^ | Molecular sieve | NR | NR | On the market | FDA approved | FAA approved |
| OxLife Liberty2 | 91% ± 4%^*^ | 44.4 dBA at setting 2 of PF^*^ | 2.5hours^*^ | -0.08 to -0.35 cmH2O | NR | NR | 2 Jan 2024 | On the market | NR | FAA approved |
| DISCOV-R | Above 82%^*^ | <54 dB^*^ | Up to 6 h for an 8-cell battery^*^ | <-0.24 cmH2O^*^ | NR | NR | June 2023 | On the market | FDA approved | FAA approved |
| Inogen Rove 6 | 90 +6/ - 3% at all settings^*^ | 39 dBA^†^ | Single Battery: up to 6.25h/ Double Battery: up to 12.75h^†^ | <0.12 cm H20^*^ | Molecular sieve | PSA | NR | On the market | FDA approved | FAA approved |
| EverGo | 89 ± 3%^§^ | <50 dB^§^ | 2-3h^§^ | -0.2 cm H20^§^ | NR | NR | NR | On the market | FDA approved | FAA approved |
| Simply Go | 90 +6/ -3%^§^ | 43 dBA at PF setting^§^ | Less than 4 h^†^ | ≤ 0.3 cm H2 O at PF setting^*^ | NR | NR | NR | On the market | FDA approved# | FAA approved |
| Zen-O | 90 +6/ -3%^†^ | 38 dB^†^ | 4h at setting 1^†^ | -0.12 cmH2O^*^ | Molecular sieve | NR | 2016 | On the market | FDA approved# | FAA approved |
| Easy Pulse TOC PM4400 series | 90 +5/ -3%^§^ | >45 dB^*^ | 2h^**^ | NR | NR | NR | NR | On the market | NR | FAA approved |
| Saros | 93 ± 3% for all flow settings^*^ | <59 dB^*^ | 2.4hours to achieve 80% capacity from a fully discharged battery^*^ | Adjustable 0.15 – 0.45 cmH2O of (negative pressure)^*^ | Zeolite | PSA,Vacuum pressure cycle | NR | On the market | FDA approved | "It meets Federal Aviation Administration (FAA) guidelines" |
| eQuinox (Model 4000) | 90 +5.5/ -3%^*^ | 45 dB at 2.0 LPM of CF^*^ | 1.5 - 3.5h^*^ | Adjustable between -0.13 - -0.4 cmH20^*^ (negative pressure). | NR | NR | NR | On the market | FDA approved | FAA approved |
| Oxlife INDEPENDENCE | 91 ± 4%^*^ | 56 dB^*^ | 1.5h^*^ | NR | NR | NR | NR | On the market | NR | FAA approved |
| 4-SLPM (Prototype) | NR | NR | NR | NR | LiLSX | PSA | NR | Not for sale | NR | NR |
| Pressure Swing Adsorption (PSA) (Prototype) | 90% at 1 LPM of CF 85% at 2 LPM of CF 75% at 3 LPM of CF 45% at 6 LPM of CF^§^ | NR | NR | NR | Zeolite | PSA | NR | Not for sale | NR | NR |
| Eclipse 5 | 90 +5.5/ -3%^*^ | 48 dBA at 3.0 LPM of CF/ 40 dBA at setting 3 of PF^*^ | 1.8 - 5.0h to achieve 80% capacity^*^ | -0.135-0.37 cm/H2O (adjustable)^*^ | NR | VPSA | 2012 | On the market | FDA approved | FAA approved |
| SOLO_2_ | 87 - 95.6%^*^ | <39dB at 2 LPM of CF/ <40dB at PF setting^*^ | 4.5h^*^ | -0.20 cm H2O^*^ | NR | NR | NR | Not for sale | FDA approved | FAA approved |
| Juno Portable Oxygen Concentrator | 91%^**^ | NR | NR | NR | NR | NR | NR | Not for sale | “This product is currently in clinical development and has not yet been approved by the FDA” | NR |

**Note:** ^*^Data reported in the product manual; ^**^Data reported in the manufacturer’s official website; ^†^Data obtained from non-manufacturer websites; ^§^Data reported in the scientific reports.

**Abbreviations:** AC, alternating current; CF, continuous flow; dBA, A-weighted decibels; DC, direct current; FAA, Federal Aviation Administration; FDA, Food and Drug Administration; LPM, liters per minute; LPMeq, liter per minute equivalent; NR, not reported; PF, pulse flow; PSA, pressure swing adsorption; VPSA, vacuum pressure swing adsorption.

Table S5 The performance of each identified liquid oxygen product

| Name | Manufacturer | Size | Weight | Transport method | Gaseous oxygen capacity | PF/CF | Setting in PF | Flow rates in CF | Maximum operating time | FDA approved |
| --- | --- | --- | --- | --- | --- | --- | --- | --- | --- | --- |
| Stroller | CAIRE | H 34.3 x W 19.1 x D 14.9cm | 3.63kg (filled) | Backpack and cart accessories available | 1026L | only CF | NR | 0.25 - 6LPM^*^ | 8.5h at 2LPM^*^ | FDA approved |
| CAIRE Spirit 300 | CAIRE | H 22.3 W 15.2 x D 11.4 cm | 1.95kg (filled) | Belt pack or Back pack | 275L | Both | 1-5 | 2LPM^*^ | 9h at 2LPM of CF^*^ | FDA approved |
| Companion 1000 | Puritan-Bennett | H 36.8 x W16.5 X D 15.5 cm | 3.7kg (filled) | Cart or Backpack | 1058L | only CF | NR | 0-4LPM^*^ | 7.6h at 2LPM^*^ | FDA approved |
| Eazy mate 6+6 PM2120 | DEHAS Medical Systems GmbH | H38.0 x W14.6 x D12.2 cm | 3.6kg (filled) | Carry bag | NR | Both | 2-6 | 0-6LPM^*^ | 6h at 2LPM of CF^*^ | FDA approved |
| FREELOX | Taema | H36.5 x L19.5 x W 19.5 cm | 3.5kg (filled) | Belt pack or cart | 830L | only CF | NR | 0-7LPM^*^ | 6h at 2LPM of CF^*^ | NR |
| Helios 300 portable | Puritan-Bennett | H26.7cm | 1.6kg (filled) | Handle or Belt pack | 308L | Both | 1-4 | 0.12-0.75L^*^ | 10h at 2LPM using an conserving module^*^ | FDA approved |

**Note:** ^*^Data reported in the product manual.

**Abbreviations:** CF, continuous flow; FDA, Food and Drug Administration; LPM, liters per minute; NR, not reported; PF, pulse flow.

Table S6 Summary of Home Oxygen Filling System

| Name of cylinder | Manufacturer | Size | Weight | Oxygen content | Cylinder Filling Times | Service Pressure | Name of Station system |
| --- | --- | --- | --- | --- | --- | --- | --- |
| iFill ML6 Cylinder | Drive DeVilbiss | NR | 2.18 kg | 165L | 90m | 2,000 ± 200 psig | iFill Personal Oxygen Station |
| iFill C Cylinder | Drive DeVilbiss | NR | 2.44 kg | NR | 2h10m | 2,000 ± 200 psig |  |
| iFill D Cylinder | Drive DeVilbiss | NR | 3.18 kg | NR | 3h35m | 2,000 ± 200 psig |  |
| HomeFill M9 Cylinder | Invacare | H38.1 x D11.1cm | 2.44kg | 245L | 2h 5m | 1800 psig | HomeFill Oxygen Compressor |
| HomeFill D Cylinder | Invacare | H53.3 x 11.1cm | 2.95kg | 425L | 3.5h | 1800 psig |  |
| UltraFill MB08 Cylinder | Philips | H 39.6cm | 1.45kg | NR | 1h 45m | 3000 psi | UltraFill  Filling station |

**Abbreviations:** NR, not reported; Psi, pounds per square inch.

Table S7 Summary of development

| Publish year | Type of publication | Type of field | Country | Developer or Applicant | Title | 10 -20 words contents | Development phase |
| --- | --- | --- | --- | --- | --- | --- | --- |
| Downsize and Portability Enhancement | | | | | | | |
| 2024 | Patent |  | AUS | ROAM TECHNOLOGIES PTY LTD | OXYGEN CONCENTRATOR | Portable/handheld APSA-based oxygen concentrator using low-pressure adsorption, deep-vacuum desorption, downsized columns, and a removable product-buffer module. | Patent |
| 2024 | Patent |  | USA | Inogen, Inc. | GAS CONCENTRATOR WITH REMOVABLE CARTRIDGE ADSORBENT BEDS | Modular portable concentrator with user-replaceable adsorbent beds and seals. | Patent |
| 2023 | Patent |  | INDIA | Nazia Parveen, et al | PORTABLE OXYGEN CONCENTRATOR WITH FAULT DETECTION | Battery-powered concentrator ensures reliable oxygen and monitoring in low-resource settings. | Patent |
| 2023 | Patent |  | INDIA | AARTHI RAMACHANDRAN, et al | PORTABLE OXYGEN CONCENTRATING DEVICE FACILITATING USER INTERACTION AND AUTOMATIC REGENERATION | Portable device generates oxygen-enriched air using nitrogen adsorption and automation. | Patent |
| 2023 | Patent |  | USA | Worldwide Health Innovations, LLC | DEVICE AND METHOD OF GENERATING AN ENRICHED GAS WITHIN A NASAL VESTIBULE | Nasal device enriches gas via molecular sieve for targeted delivery. | Patent |
| 2023 | Patent |  | USA | WEARAIR VENTURES, INC. | EFFICIENT ENRICHED OXYGEN AIRFLOW SYSTEMS AND METHODS | Portable concentrator delivers low-concentration, high-volume oxygen for efficiency. | Patent |
| 2023 | Review article (Narrative review) | Medicine or Health | INDIA | Mody University of Science and Technology | Recent trends in the nanozeolites-based oxygen concentrators and their application in respiratory disorders. | There are several procedures by which the efficiency of such oxygen concentrators and the purity of oxygen can be achieved. One such process is the application of nanozeolites | N/A |
| 2022 | Patent |  | USA | Vbox, Incorporated | REMOVABLE CARTRIDGE FOR OXYGEN CONCENTRATOR | Removable cartridge uses adsorbent bed to separate oxygen from air. | Patent |
| 2022 | Patent |  | USA | ROAM TECHNOLOGIES PTY LTD | SYSTEMS AND METHODS FOR PROVIDING CONCENTRATED OXYGEN TO A USER | Portable concentrator uses zeolite bed to extract oxygen from air. | Patent |
| 2022 | Patent |  | USA | WEARAIR VENTURES, INC. | Efficient enriched oxygen airflow systems and methods | POC switches delivery modes and oxygen concentration based on conditions. | Patent |
| 2022 | Patent |  | USA | Oxygenium Ltd. | Portable System for the Production of Oxygen | Portable system generates breathable oxygen from hydrogen peroxide reaction. | Patent |
| 2021 | Scientific paper | Engineering | USA | Texas A&M University | Flexible oxygen concentrators for medical applications. | Flexible PSA/PVSA MOCs achieve 95% oxygen with CPS integration. | NR |
| 2013 | Scientific paper | Engineering | TURKEY | Gazi University | Realization of a high-performance oxygen concentrator system with on-line monitoring and off-line reporting abilities | In this section, in order to achieve a high-purity oxygen from the system output, a new structure based on the principle of equal pressure application to the surface of molecular sieves is used in the absorption columns of OC apparatus. | developed "prototype" |
| Improving or Optimizing the Oxygen Generation Process | | | | | | | |
| 2024 | Patent |  | CHINA | Breathe Technoloogies, Inc. | O2 CONCENTRATOR WITH SIEVE BED BYPASS AND CONTROL METHOD THEREOF | PSA oxygen concentrator using a controllable sieve-bed bypass to stabilize purity during load changes via regulated valve feedback. | Patent |
| 2024 | Scientific paper | Engineering | THAILAND | King Mongkut’s University of Technology | High-Efficiency Oxygen Production through Autotuned Pressure Swing Adsorption Technology | Four-cycle PSA oxygen separator using zeolite 5A and 13X with autotuned pressure–time control; maintained oxygen purity ≥95% and achieved peak purity 95.6% with recovery within 3 minutes after air-composition disturbances. | NR |
| 2024 | Scientific paper | Engineering | INDIA | Electronics and Telecommunication Vishwakarma Institute of Technology | Design and Performance Evaluation of a Pressure Swing Zeolite Oxygen Concentrator | PSA-based oxygen concentrator prototype using compressor, HEPA filter, pressure regulator, precharge valves, and zeolite molecular sieve beds | NR |
| 2024 | Scientific paper | Engineering | INDIA | Institute of Technology Pune | Design and Performance Evaluation of a Pressure Swing Zeolite Oxygen Concentrator | PSA concentrator offers portable, cost-effective oxygen with 88.5% purity. | NR |
| 2024 | Patent |  | USA | MyAutO2, LLC | AUTOMATED OXYGEN THERAPY DEVICE AND RELATED METHODS | Device adjusts oxygen flow based on predicted patient oxygen saturation level. | Patent |
| 2023 | Scientific paper | Medicine or Health | MOROCCO | Abdelmalek Essaadi University | Small-scale medical oxygen production unit using PSA technology: modeling and sensitivity analysis. | The developed mathematical model and sensitivity analysis have provided valuable insights into the design and operation parameters of the PSA system, aiming to optimise oxygen purity, production capacity, and energy consumption. | developing prototype |
| 2023 | Patent |  | USA | Telesair, Inc. | Oxygen Supply Device With FiO2 Control | Device blends and conditions oxygen with alternating adsorption units control. | Patent |
| 2023 | Scientific paper | Engineering | INDONESIA | SEEI ITB | Design of an Embedded Controller and Optimal Algorithm of PSA for a Novel Medical Oxygen Concentrator | Optimized PSA concentrator delivers 95% oxygen using six-stage algorithm. | NR |
| 2023 | Scientific paper | Engineering | INDIA | N/A | Development of Portable Oxygen Concentrator- A Review | Optimized PSA systems use real-time control and GPRS error transmission. | N/A |
| 2022 | Scientific paper | Engineering | PAKISTAN | NED University of Engineering & Technology | Oxygen Concentrator Design: Zeolite Based Pressure Swing Adsorption | Results show LiX is the most suitable with better nitrogen to oxygen selectivity ratio with a flow rate of 5LPM. | developed "prototype" |
| 2021 | Scientific paper | Engineering | CHINA | Dalian Institute of Chemical Physics, et al | Experimental and Numerical Analysis on the Enhanced Separation Performance of a Medical Oxygen Concentrator through Two-Bed Rapid Pressure Swing Adsorption | RPSA system with Li-LSX achieves 94-95% oxygen in 14s cycles. | NR |
| 2019 | Review article (Narrative review) | Engineering | USA |  | Medical oxygen concentrators: a review of progress in air separation technology | Advanced LiX adsorbents and pulse-flow strategies enhance portable oxygen concentrators. | N/A |
| 2022 | Patent |  | USA | Inova Labs, Inc. | SYSTEM AND METHOD OF DESORBING NITROGEN FROM PARTICLES | Oxygen concentrator includes features extending adsorbent material's useful lifetime. | Patent |
| 2022 | Review article (Narrative review) | Engineering | INDIA | Jerusalem college of Engineering | Cost efficient oxygen concentrator with PSA technology | Cost-effective PSA concentrator delivers 94.7% oxygen at low flow. | NR |
| 2022 | Patent |  | USA | Jeff Majdali | Oxygen therapy administration methods and related apparatus | High-flow oxygen therapy adjusts flow to match inspiratory demand. | Patent |
| Remote Control | | | | | | | |
| 2025 | Scientific paper | Medicine or Health | DENMARK | Copenhagen University Hospital | Optimised oxygenation improves functional capacity during daily activities in patients with COPD on long-term oxygen therapy: a randomised crossover trial | Closed-loop automated oxygen titration using O2matic device and Bluetooth wrist pulse oximeter; flow adjusted 0–8 L/min targeting SpO₂ 90–94% during ADL; improved ADL test time by median 38 s, increased time within target saturation, and reduced dyspnoea compared with fixed oxygen flow. | NR |
| 2025 | Scientific paper | Engineering | INDIA | Sphoorthy Engineering College | IoT-Based Adaptive Oxygen Regulation System with Artificial Neural Networks for Personalized Care | IoT-enabled adaptive oxygen regulation system using ANN to predict ideal oxygen flow based on SpO2, respiratory rate, temperature, humidity, and activity; real-time adjustment through feedback loop with cloud-based processing and sensor network. | NR |
| 2025 | Scientific paper | Engineering | INDIA | CSIR-Central Mechanical Engineering Research Institute | RESPIPulse: Machine learning assisted sensory device for pulsed mode delivery of oxygen bolus using surface electromyography (sEMG) signals | Pulsed-mode oxygen bolus device using sEMG-based detection of breathing phases with machine-learning-supported exhalation prediction, integrated airflow sensing, and solenoid-valve control; user testing demonstrated reliable phase identification and substantial oxygen conservation. | NR |
| 2024 | Scientific paper | Engineering | CHINA | Northwestern Polytechnical University | Portable oxygen breathing apparatus integrated with biosensors: Enabling intelligent monitoring and optimal oxygen provision for biomechanical homeostasis | Intelligent portable oxygen breathing apparatus (IPOBAB) integrating biosensors (IMU, SpO₂, heart rate) and machine learning (Dynamic Gradient Boosting Machine) to classify exertion levels and automatically adjust oxygen flow. | NR |
| 2024 | Scientific paper | Engineering | SPAIN | Universitat Politècnica de Catalunya | Innovative Predictive Approach towards a Personalized Oxygen Dosing System | Edge-based AI architecture using patient historical data and activity context to proactively predict individualized oxygen dosing needs; pilot test on five LTOT patients using SpO2 and heart rate vital-sign streams. | NR |
| 2024 | Scientific paper | Engineering | INDIA | Department of Electrical Engineering Indian Institute of Technology Ropar | A Pulse Oximeter and a Controller Designed for Automatic Regulation of Oxygen Concentrators | Pulse-oximeter PCB prototype integrated with oxygen concentrator; closed-loop automatic regulation of flow rate using SpO2-derived PPG signals and Simulink-based valve control. | developed "prototype" |
| 2024 | Scientific paper | Engineering | INDIA | Division of Biomedical Engineering Karunya Institute of Technology and Sciences | A Novel IoT-Enabled Oxygen Delivery System with Neural Network-based Predictive Control | Intelligent portable oxygen concentrator using neural-network model predicts optimal oxygen flow rate based on SpO2, HR, RR, and temperature; supports IoT-based remote control and real-time physiological monitoring. | NR |
| 2024 | Scientific paper | Engineering | INDIA | Indian Institute of Technology Ropar | A Pulse Oximeter and a Controller Designed for Automatic Regulation of Oxygen Concentrators | System auto-adjusts oxygen flow using SpO2 data and Simulink simulation. | developing "prototype" |
| 2023 | Review article (Narrative review) | Engineering | INDONESIA | School of Electrical Engineering and Informatics | Design and implementation system of mobile oxygen concentrator and telemedicine for comprehensive treatment of SpO2 | Smart Oxycon system with telemedicine improves SpO2 and remote monitoring. | developed "prototype" |
| 2023 | Patent |  | USA | Telesair, Inc. | Method and system of remote control and remote monitor in treating respiratory patients | Oxygen device allows local or remote user control via instructions. | Patent |
| 2023 | Scientific paper | Engineering | USA | Pratt School of Engineering, Duke University, Durham, NC, USA | Design and Development of a Novel System for Remote Control of Stationary Oxygen Concentrator Flow Rate. | Remote oxygen control achieved with high accuracy and home-range usability. | developed "prototype" |
| 2022 | Patent |  | USA | Telesair, Inc. | Facilitating remote control of oxygen supply | Oxygen device supports local and remote user control operation modes. | Patent |
| 2020 | Scientific paper | Engineering | SPAIN | INiBICA | A Prototype of Intelligent Portable Oxygen Concentrator for Patients with COPD Under Oxygen Therapy | Activity-based oxygen adjustment uses IMU and logistic regression classifier. | developed "prototype" |
| 2020 | Scientific paper | Engineering | SPAIN | INiBICA | Automated Home Oxygen Delivery for Patients with COPD and Respiratory Failure: A New Approach. | Intelligent POC adjusts oxygen flow by classifying patient activity level. | developed "prototype" |
| 2016 | Scientific paper | Engineering | CANADA | NED University of Engineering & Technology | Portable Automated Oxygen Administration System for hypoxaemic patients | The prototype model measures the SpO2 and consequently adjusts the amount of supplemental oxygen that is delivered to the patient accordingly using simple oxygen administration algorithm. | developed "prototype" |
| 2015 | Scientific paper | Engineering | INDIA | Rajalakshmi Engineering College | An indegenous oxygen dosing device to conserve oxygen using patient monitoring system | Device adjusts oxygen dose using respiration signal and SpO2 data. | developing "prototype" |
| 2013 | Review article (Narrative review) | Medicine or Health | FRANCE | N/A | Optimal oxygen titration in patients with chronic obstructive pulmonary disease: a role for automated oxygen delivery?. | These closed-loop oxygen delivery systems have the potential to reduce medical error, improve morbidity and mortality, and reduce health care costs. | N/A |
| Other | | | | | | | |
| 2024 | Patent |  | USA | Belluscura LLC | SYSTEMS AND METHODS FOR IMPROVING PATIENT HEALTH | System adjusts oxygen therapy using consciousness, device data, and sleep history. | Patent |
| 2024 | Patent |  | USA | Ravit BARKAMA | SYSTEMS AND METHODS FOR SUPPLEMENTAL OXYGEN DELIVERY | System personalizes oxygen delivery by analyzing sensors and subject data. | Patent |
| 2023 | Patent |  | WIPO | ODA OSSIGENO DALL'AMBIENTE S.R.L. | MONITORING DEVICE OF AN OXYGEN CONCENTRATOR | Device calculates real oxygen purity using flow, purity, and environment data. | Patent |
| 2023 | Patent |  | WIPO | VENTEC LIFE SYSTEMS, INC. | SYSTEMS AND METHODS FOR GENERATING CONCENTRATED OXYGEN | User-replaceable media beds generate concentrated oxygen from ambient air. | Patent |
| 2023 | Patent |  | USA | Breathe Technologies, Inc. | O2 CONCENTRATOR WITH SIEVE BED BYPASS AND CONTROL METHOD THEREOF | Oxygen concentrator uses bypass valve for ventilator-controlled oxygen delivery. | Patent |
| 2023 | Patent |  | USA | Inogen, Inc. | Concentrator with electronic handheld remote delivery device | Remote system adjusts oxygen flow using pneumatic feedback and control settings. | Patent |
| 2023 | Patent |  | USA | Neal Ramchandani | AUTOMATED OXYGEN MONITORING SYSTEMS TO TITRATE SUPPLEMENTAL OXYGEN, ARRANGEMENTS INCORPORATING THE SAME, AND METHODS THEREFOR | Automated system adjusts oxygen delivery based on patient saturation sensor. | Patent |
| 2023 | Patent |  | INDIA | TEIJIN PHARMA LIMITED | OXYGEN CONCENTRATION DEVICE | Device enables secure, easy replacement of adsorption cylinder with intersecting axes. | Patent |
| 2022 | Patent |  | USA | Volker Karel Espinoza Torres | INTELLIGENT AUTOMATIC OXYGEN THERAPY SYSTEM | AI-driven system adjusts oxygen dosage using SpO2 and sensor data. | Patent |
| 2022 | Patent |  | USA | Seabeck Holdings, LLC | PULSED OXYGEN SYSTEM AND PROCESS | System delivers microburst oxygen using optical sensor and solenoid control. | Patent |
| 2022 | Patent |  | USA | Telesair, Inc. | Integrated oxygen supply device | Device stores generated oxygen and releases it based on breathing patterns. | Patent |
| 2021 | Scientific paper | Engineering | THAILAND | King Mongkut's Institute of Technology Ladkrabang | Pressure Swing Absorption Oxygen Concentrator equipped with Remote Monitoring Pulse Oximeter | Experiment has shown that our emergency low-cost oxygen concentrator can supply oxygen with an 85% purity rate. | developing prototype |

**Abbreviations:** AI, artificial intelligence; CF, IMU, inertial measurement unit; LiLSX, lithium-exchanged low silica X-type zeolite; LPM, liters per minute; NR, not reported; OC, oxygen concentrator; PSA, pressure swing adsorption; PVSA, pressure vacuum swing adsorption; RPSA, rapid pressure swing adsorption; SpO₂, peripheral capillary oxygen saturation; WIPO, World Intellectual Property Organization.
